# Transcriptome of the inflorescence meristems of the biofuel plant *Jatropha curcas* treated with cytokinin

**DOI:** 10.1186/1471-2164-15-974

**Published:** 2014-11-17

**Authors:** Bang-Zhen Pan, Mao-Sheng Chen, Jun Ni, Zeng-Fu Xu

**Affiliations:** Key Laboratory of Tropical Plant Resources and Sustainable Use, Xishuangbanna Tropical Botanical Garden, Chinese Academy of Sciences, Kragujevac, Yunnan, Menglun 666303 People’s Republic of China; School of Life Sciences, University of Science and Technology of China, Hefei, Anhui 230027 People’s Republic of China

**Keywords:** Biofuel, Physic nut, Cytokinin, 454 Sequencing, Flower, Cell division

## Abstract

**Background:**

*Jatropha curcas*, whose seed content is approximately 30–40% oil, is an ideal feedstock for producing biodiesel and bio-jet fuels. However, *Jatropha* plants have a low number of female flowers, which results in low seed yield that cannot meet the needs of the biofuel industry. Thus, increasing the number of female flowers is critical for the improvement of *Jatropha* seed yield. Our previous findings showed that cytokinin treatment can increase the flower number and female to male ratio and also induce bisexual flowers in *Jatropha*. The mechanisms underlying the influence of cytokinin on *Jatropha* flower development and sex determination, however, have not been clarified.

**Results:**

This study examined the transcriptional levels of genes involved in the response to cytokinin in *Jatropha* inflorescence meristems at different time points after cytokinin treatment by 454 sequencing, which gave rise to a total of 294.6 Mb of transcript sequences. Up-regulated and down-regulated annotated and novel genes were identified, and the expression levels of the genes of interest were confirmed by qRT-PCR. The identified transcripts include those encoding genes involved in the biosynthesis, metabolism, and signaling of cytokinin and other plant hormones, flower development and cell division, which may be related to phenotypic changes of *Jatropha* in response to cytokinin treatment. Our analysis indicated that *Jatropha* orthologs of the floral organ identity genes known as ABCE model genes, *JcAP1,2, JcPI*, *JcAG*, and *JcSEP1,2,3*, were all significantly repressed, with an exception of one B-function gene *JcAP3* that was shown to be up-regulated by BA treatment, indicating different mechanisms to be involved in the floral organ development of unisexual flowers of *Jatropha* and bisexual flowers of *Arabidopsis*. Several cell division-related genes, including *JcCycA3;2*, *JcCycD3;1*, *JcCycD3;2* and *JcTSO1*, were up-regulated, which may contribute to the increased flower number after cytokinin treatment.

**Conclusions:**

This study presents the first report of global expression patterns of cytokinin-regulated transcripts in *Jatropha* inflorescence meristems. This report laid the foundation for further mechanistic studies on *Jatropha* and other non-model plants responding to cytokinin. Moreover, the identification of functional candidate genes will be useful for generating superior varieties of high-yielding transgenic *Jatropha*.

**Electronic supplementary material:**

The online version of this article (doi:10.1186/1471-2164-15-974) contains supplementary material, which is available to authorized users.

## Background

The ever-decreasing crude oil reserves are insufficient to satisfy the increasing demand for petroleum as a transportation and heating fuel, and petroleum consumption also pollutes the environment. Liquid biofuels from plants and microalgae may help solve these problems. *Jatropha curcas* (hereafter referred to as *Jatropha*), a perennial deciduous shrub belonging to the family Euphorbiaceae whose seed content is approximately 30–40% oil, is an ideal feedstock for producing biodiesel and bio-jet fuels [1–3]. Because the quality parameters of *Jatropha* biodiesel are within the European EN 14214 specification and the emission parameters of sulfur and particulate matter are 80% lower than those of mineral diesel [4], *Jatropha* is emerging as a potential biofuel plant.

However, currently the seed yield of *Jatropha* is poor and insufficient for the biodiesel industry [5, 6]. Recently, researchers reported that applying plant growth regulators to *Jatropha* can improve seed yield [7–9]. Our previous study found that applying benzyladenine (BA, a synthetic cytokinin) to the inflorescence meristems of *Jatropha* significantly increased the flower number and the female to male flower ratio, which contributed to an increase in seed yield [10]. Cytokinin is involved in plant development and growth and can be used to regulate many aspects of plant development in both practical and theoretical studies [8–16]. Endogenous levels of cytokinin content increased in *Polianthes tuberosa* and *Litchi chinensis* when they began to flower [17, 18]. In *Arabidopsis*, cytokinin treatment correlated with early flowering [19, 20]. Transgenic *Arabidopsis* plants overexpressing *CYTOKININ OXIDASE* (*CKX*), which degrades cytokinins, flowered late [21]. These studies indicated that cytokinin stimulates flowering in these plants. Additionally, cytokinin increased the flower number in *Arabidopsis* [13] and induced an aberrant floral phenotype including an increased number of flower organs [12, 22]. Exogenous cytokinin application and accumulation of endogenous cytokinin increased the flower number in several species [10, 13, 23–25]. In addition, the flower sex of *Vitis vinifera* [26, 27], *Luffa cylindrical* [28], *Momordica charantia* [29] and *Pinus densiflora* [30] was also affected by exogenous cytokinins.

It is not economically viable to improve *Jatropha* seed yield by exogenous application of BA in large scale plantation. Generating transgenic *Jatropha* plants with increased female and/or bisexual flower number is critical for improving seed yield. The initial step of transgenic *Jatropha* study is to identify functional genes. Therefore, the identification of genes involved in flower development following BA treatment and the characterization of their expression profiles are two important prerequisites. Currently, next-generation sequencing technologies make it relatively inexpensive to study the transcriptome of a particular organism or tissue to gain insight into biological processes. In *Cucumis stativus*, the transcriptomes of flowers of different sexes were sequenced to determine the molecular mechanisms of plant sex determination [31]. To study the genetic control of *Fagopyrum* floral development*,* the floral transcriptomes of two species that have the ability to self-pollinate, in contrast to the common *Fagopyrum*, were characterized [32]. The floral transcriptome was also sequenced to evaluate self-incompatibility in *Ziziphus celata*, a highly endangered plant [33]. Recently, transcriptome analysis was used to investigate global expression patterns of phytohormone-regulated transcripts in tomato leaves and roots [34, 35]. Therefore, transcriptome sequencing is a proven strategy for expression profiling of genes involved in various processes in plants.

Although the *Jatropha* genome has been sequenced by a combination of the conventional Sanger method and next-generation multiplex sequencing methods [36, 37], most of the molecular studies on *Jatropha* have focused on lipid metabolism in seeds. Global analysis of gene expression profiles in developing and germinating seeds were performed to assess differential gene expression and to discover genes involved in lipid metabolism [38–45]. For high-throughput discovery of novel *Jatropha* genes, de novo assembly and transcriptome analysis of different tissues of *Jatropha* were performed [46]. However, molecular studies on flower development and/or flower developmental responses to phytohormone treatment in *Jatropha* are scarce.

Given that BA increased flower number and the female to male ratio and induced bisexual flowers in *Jatropha* [10], we conducted a time course study of gene expression profiles in inflorescence meristems of *Jatropha* exposed to BA. One 454 sequencing run was performed that generated a total of 294.6 Mb of transcript sequences. Differentially expressed genes involved in the biosynthesis, metabolism, and signaling of cytokinin and other plant hormones, flower development and cell division, which may be related to the phenotypic changes of *Jatropha* in response to cytokinin treatment, were further analyzed.

We expect that characterization of the transcriptome of *Jatropha* inflorescence meristems treated with BA will contribute not only to genetic engineering and breeding of *Jatropha* but will also provide insight into the mechanism of how cytokinin affects the flower development of *Jatropha* and other non-model plants.

## Results and discussion

### Effects of BA on *Jatropha*flower development

**Figure 1:**
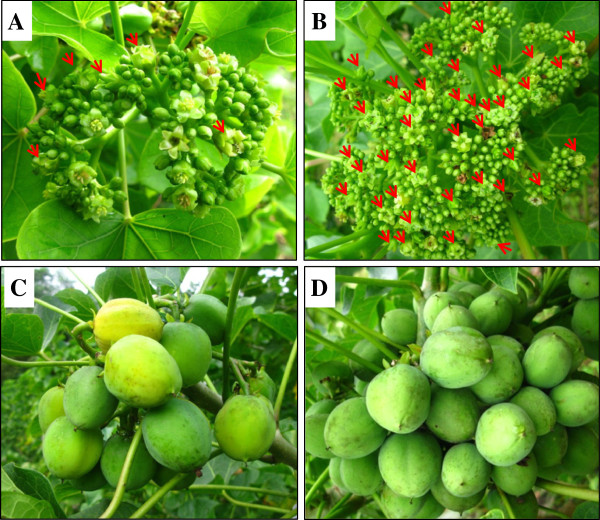
**Effects of BA on flower development and fruiting of** ***Jatropha*** **.** Inflorescence **(A, B)** and infructescence **(C, D)** from control plants **(A, C)** and BA-treated plants **(B, D)**. Female flowers are marked with red arrows in **A** and **B**.

To quantitatively apply BA to inflorescence meristems of *Jatropha*, absorbent cotton containing a BA solution was used rather than hand sprayers as in previous studies [10]. In line with our previous work [10], BA application to the inflorescence meristems of *Jatropha* resulted in a significant increase in the total flower number and the female flower number of each inflorescence (Figure 1A,B, Additional file 1: Figure S1A). In addition to promoting the number of normal female and male flowers, BA treatment also induced bisexual and asexual flowers (Additional file 1: Figure S1). As the number of female flowers is one of the most important factors affecting fruiting, the increased female flower number and the newly induced bisexual flowers contributed to more fruits than the control (Figure 1C,D, Additional file 1: Figure S1A).

### 454 sequencing and transcriptome assembly

**Figure 2:**
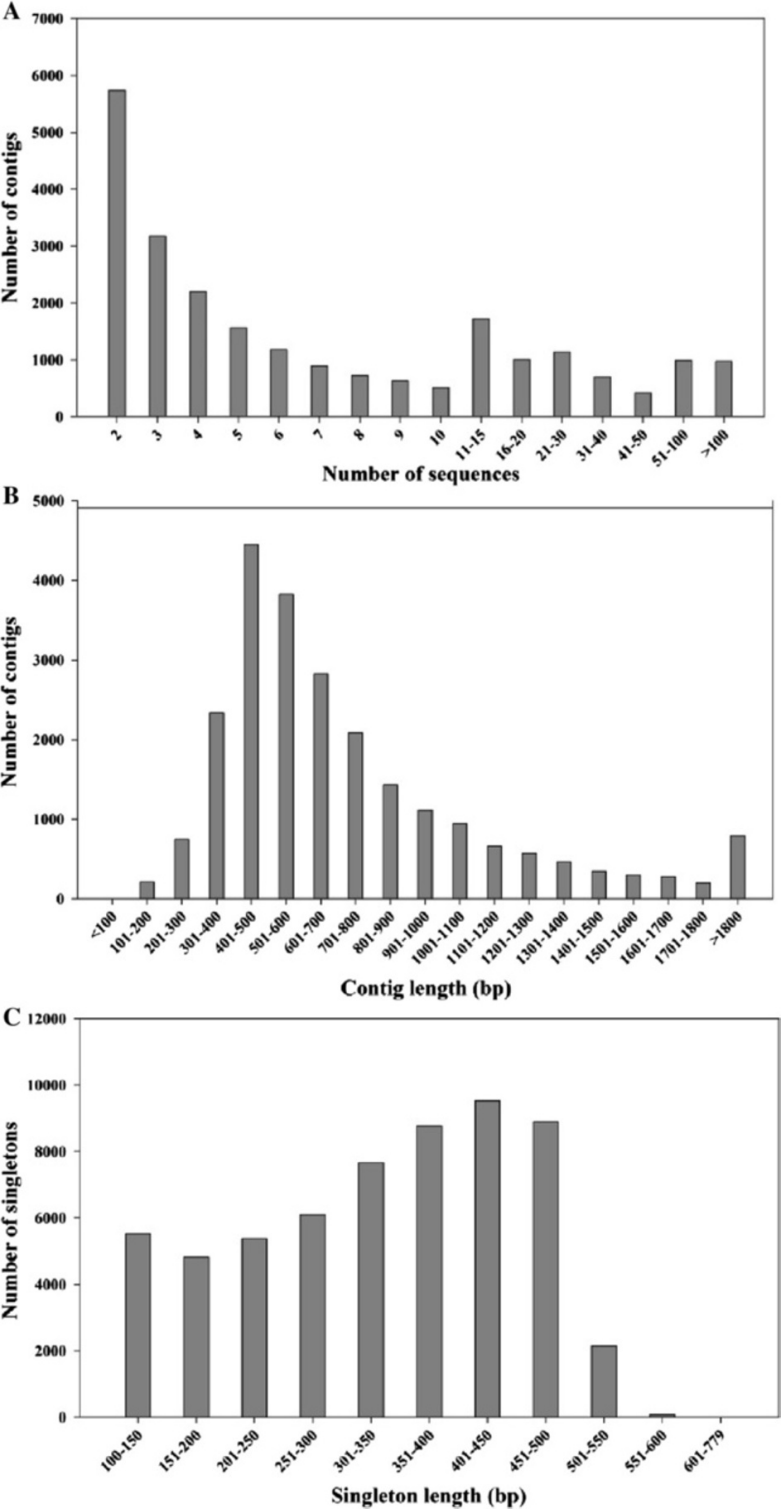
**Distribution of the number of reads per contig and the length distribution of the contigs and singletons. (A)** Distribution of the sequences in the contigs; **(B)** Length distribution of the contigs; **(C)** Length distribution of the singletons.

For 454 transcriptome sequencing of *Jatropha* inflorescence meristems, four cDNA libraries were constructed from inflorescence meristems collected before BA treatment (Control), BA treatment for 2 hours (T), 4 hours post BA treatment (T +4 H) and 22 hours post BA treatment (T +22 H), respectively. Each cDNA library was used in a one-quarter run on a 454 GS FLX Titanium instrument, which produced a total of 839,205 raw reads (Table 1) that were handled by a 454 GS FLX system to cut off the adapters and low quality bases. Then, all raw reads were subjected to processing using an in-house-developed program to remove the low-quality reads. This procedure yielded 703,755 high-quality reads ranging from 100 bp to 790 bp, with an average length of 364 bp (Table 1). A total of 644,835 reads, corresponding to 91.63% of the high-quality reads, were assembled by the CAP3 program [47] using default parameters with an overlap of >40 bp and an identity of 90%. This analysis gave rise to 23,591 contigs with an average length of 740 bp, and 58,920 reads were identified as singletons (Table 1). Approximately 75.68% of the contigs were assembled from three or more reads. The distribution of the number of reads per contig, the length distribution of the contigs and the singletons are presented in Figure 2.

**Table 1:** **Characteristics of raw data and assembly summary**

	0 HAT	2 HAT	6 HAT	24 HAT
Raw sequencing reads	200,913	201,598	248,674	188,020
Total number of raw read		839,205		
Average length of raw read		351 bp		
High quality reads	182,722	172,682	181,699	166,652
Total number of high quality read		703,755		
Average length of high quality read		364 bp		
Length range of high quality read		100 bp - 790 bp		
Total number of singleton		58,920		
Average length of singleton		334 bp		
Total number of contig		23,591		
Assembled reads (of total number of high quality read)		644,835 (91.63%)		
Average length of contig		740 bp

### Functional annotation

Open reading frames (ORFs) were identified using an in-house developed program based on ‘GetORF’ from EMBOSS, which predicted CDS of 81,736 unigenes (Table 2). The annotations of the unigenes were identified through a BLAST search against the Swiss-Prot and GenBank databases with an E-value cut-off of 1e-3, which resulted in approximately 78.12% (18,429) of the contigs and approximately 48.50% (28,356) of the singletons with significant matches (Table 2). Approximately 10,727 contigs and 12,864 singletons were assigned to clusters of orthologous groups for eukaryotic complete genomes (KOG). Meanwhile, gene ontology (GO) terms were assigned to 8,148 contigs and 11,030 singletons. We also mapped the sequences of all of the unigenes to the *Jatropha* genome sequences constructed by Sato et al. [36] and upgraded by Hirakawa et al. [37], which gave rise to 18,504 contigs and 28,848 singletons with significant matches. There are still 5,087 contigs (21.56%) and 29,622 singletons (50.66%) with no assigned function, which represent a source for gene discovery (Table 2).

**Table 2:** **Transcriptome annotation summary**

	Contigs	Singletons	Total unigenes
Number	23,591	58,920	82,511
Predicted CDS	23,576	58,160	81,736
Average CDS length	740 bp	549 bp	604 bp
Maximum CDS length	1,230 bp	564 bp	1230 bp
CDS with known function	18,429	28,356	46,785
CDS with KOG assignment	10,727	12,864	23,591
CDS with GO classification	8,148	11,030	19,178
Unigene sequences mapped to *Jatropha* genome	18,504	28,848	47,352

CDS: coding sequence; KOG: eukaryotic orthologous groups; GO: gene ontology.

Functional annotation of CDSs was performed by searching against NCBI non-redundant protein database and KEGG protein database using BLASTP with E value 1e-3. Then the top hit protein (with the highest bit score) was chosen to count its origin (encoded by what organism). The distribution of sequences from various species closely related to those found in the *Jatropha* inflorescence meristem transcriptome is shown in Additional file 2: Table S1. More than half (58.4%) of *Jatropha* sequences are similar to sequences found in the close related castor bean (*Ricinus communis*) genome, which is consistent with the analysis of whole genome sequences of *Jatropha* [36, 37]. In contrast, the percentage of *Jatropha* sequences with top hit to sequences of the model plant *Arabidopsis* is as low as 0.81% (Additional file 2: Table S1). Given these results, studying the transcriptomes of *Jatropha* inflorescence meristems with and without BA treatment may shed light on the molecular mechanisms of cytokinin effects on the flower development of castor bean and other non-model plants.

### Transcript clustering by expression signatures

**Figure 3:**
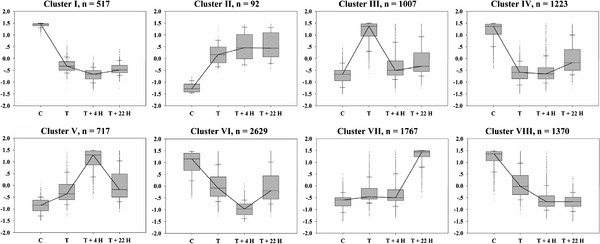
**Clustering of differentially expressed genes in the transcriptome of** ***Jatropha*** **inflorescence meristems treated with BA.** N is the number of transcripts found in each cluster. Cluster I and II contain cytokinin-repressed or induced genes at all three time points, respectively; Cluster III, V and VII contain cytokinin-induced genes at T, T +4 H and T +22 H, respectively; Cluster IV, VI and VIII contain cytokinin-repressed genes at T, T +4 H and T +22 H, respectively. A number of differentially expressed genes were included in more than one cluster according to their expression profiles. The y-axis represents the normalized expression value. The expression value of each gene was *Z*-score (mean centered, with standard deviations as the unit, p <0.05). T: BA treatment for 2 hours, T +4 H: 4 hours post BA treatment, T +22 H: 22 hours post BA treatment.

To analyze the critical cellular processes in inflorescence meristems of *Jatropha* following BA treatment, we grouped the transcripts according to their expression patterns across four samples into eight clusters (Figure 3, Additional file 3: Table S2). Cluster I contains 517 transcripts down-regulated by BA treatment at T, T +4 H and T +22 H. Cluster II has 92 transcripts up-regulated by BA treatment at T, T +4 H and T +22 H. Cluster III is composed of 1,007 transcripts that are abundant at T, Cluster V is composed of 717 transcripts that are abundant at T +4 H and Cluster VII is composed of 1767 transcripts that are abundant at T +22 H. Conversely, Cluster IV is composed of 1,223 transcripts that are down-regulated at T, Cluster VI is composed of 2,629 transcripts that are down-regulated at T +4 H and Cluster VIII is composed of 1,370 transcripts that are down-regulated at T +22 H. A number of differentially expressed genes were included in more than one cluster according to their expression profiles. Thus although there are more than 9,000 genes presented in the Figure 3, there are only 5,871 genes in total expressed differentially following cytokinin treatment actually. These results revealed that there are more down-regulated genes than up-regulated genes after BA treatment. Rashotte et al. [48] found that more genes are down-regulated by BA than by zeatin treatment and inferred that some genes may be specifically down-regulated by BA that are not actually regulated by cytokinin. A recent transcriptome analysis of cytokinin responses in tomato leaves also showed that there were more repressed genes than induced genes early (2 h) in the response to BA treatment [34].

Compared to *Arabidopsis* orthologs, a number of *Jatropha* genes showed different expression patterns in inflorescence meristems exposed to cytokinin. The difference may result from different treatment methods and/or different concentrations of BA used for treatment. Most treatments of *Arabidopsis* are on seedlings rather than on inflorescence meristems employed in this study for *Jatropha*. And the concentration of BA usually was less than 20 μM in treatments of *Arabidopsis* [49], whereas the concentration of BA used in this study was 1 mM.

### Gene ontology enrichment analysis of eight clusters

The gene ontology (GO) enrichment analysis of the genes in each cluster shown in Figure 3 identified the biological processes and molecular functions that characterize each cluster using DAVID (the Database for Annotation, Visualization and Integrated Discovery) [50, 51] (Table 3). This analysis revealed that the transcripts in Cluster I, which were down-regulated by BA treatment at T, T +4 H and T +22 H, were mainly involved in normal cell development, catabolic processes, DNA replication and carbohydrate metabolism. Transcripts up-regulated by BA in all treated samples in Cluster II involved in secondary metabolism (aromatic compounds and glutamine), nitrogen biosynthetic processes, positive regulation of signal transduction and sensory perception of light stimulus. These results coincide with previous findings that cytokinin-regulated genes are involved in glutamine and nitrogen metabolism [52] and that cytokinin-induced transcriptional and morphological effects, which mimic light effects, can be caused by exogenous application or overproduction of endogenous cytokinins [53–55]. In addition, Cluster II also showed a significant representation of transcripts related to the activities of receptors and molecular and signal transducers. Previous studies provided evidence that plants perceive and respond to cytokinins through a multistep phosphorelay pathway similar to the bacterial two-component system. The receptors and type-A response regulators are induced by cytokinin [56–58]. The transcripts at T, T +4 H and T +22 H (Cluster III, V and VII) are predominantly involved in DNA replication, fertilization, secondary metabolism, regulation of cell size, translation, hormone secretion and transcription initiation. Genes associated with activities of base pairing with mRNA, ion and RNA binding, triplet codon-amino acid adaptors, some peptidases, transferases and translation regulators were also enriched in these clusters. In *Arabidopsis*, genes respond to cytokinin after 15 or 30 min treatment were considered as early cytokinin-regulated genes, which are categorized into gene groups of secondary metabolism, signal transduction, and transcription factors [52, 59]. In this study, after BA treatment for 2 hours, most of BA-regulated genes are categorized into secondary metabolism and DNA replication (Cluster III, Table 3). Transcripts with low expression at one time point in Clusters IV, VI and VIII were associated with cellular responses to hormone stimulus and signaling, regulation of cell activity, secondary metabolism (carboxylic acid, glucose, arginine, aromatic compounds and nitrogen compounds), and circadian rhythm. Moreover, genes encoding proteins with receptor activity, signal transduction, and mental iron binding were also enriched in these clusters (Table 3).

**Table 3:** **GO enrichment analysis of differential transcripts in eight clusters (p <0.05)**

Cluster	Biological processes	Molecular function
I	axon extension involved in development	__
	developmental cell growth	
	ubiquitin-dependent protein catabolic process	
	DNA replication	
	carbohydrate metabolic process	
II	cellular aromatic compound metabolic process	receptor activity
	glutamine family amino acid metabolic process	molecular transducer activity
	nitrogen compound biosynthetic process	signal transducer activity
	positive regulation of signal transduction	
	sensory perception of light stimulus	
III	DNA replication	base pairing with mRNA
	carboxylic acid metabolic process	sodium ion binding
	cellular ketone metabolic process	RNA binding
	fertilization	triplet codon-amino acid adaptor activity
	long-chain fatty acid metabolic process	
IV	cellular response to hormone stimulus	receptor activity
	hormone-mediated signaling	signal transducer activity
	negative regulation of cell death	transmembrane receptor activity
	regulation of cell activation	ferric iron binding
		fatty acid binding
V	transcription initiation	threonine-type endopeptidase activity
	female pregnancy	sodium:hydrogen antiporter activity
	regulation of cell size	histone acetyltransferase activity
	regulation of hormone secretion	lysine N-acetyltransferase activity
	calcium ion homeostasis	monovalent cation:hydrogen
		antiporter activity
VI	carboxylic acid catabolic process	hormone binding
	cell death	transmembrane receptor activity
	gene silencing by RNA	fatty acid transporter activity
	glucose catabolic process	copper ion binding
	glycolysis	signal transducer activity
	positive regulation of signal transduction	
VII	translational initiation in response to stress	translation regulator activity
	negative regulation of translation	protein transporter activity
	glutathione metabolic process	metallopeptidase activity
	antigen processing and presentation of exogenous antigen	translation initiation factor binding
		peptidase activity
VIII	arginine metabolic process	cadmium ion binding
	cellular aromatic compound metabolic process	hormone binding
	circadian rhythm	receptor activity
	nitrogen compound biosynthetic process	signal transducer activity
	positive regulation of cell differentiation	
	signal transduction	

The enriched terms represented critical biological processes in inflorescence meristems treated with BA and indicated the cross-talk of cytokinin signaling with other biological processes, such as circadian rhythms [14, 60], glucose catabolism and nitrogen compound biosynthesis [52, 61].

### Metabolic pathway analysis

DAVID analysis also revealed the enriched pathways associated with the transcripts in each cluster (Table 4). Genes that showed a decrease in transcript abundance in response to BA in Clusters I, IV, VI and VIII were associated with apoptosis, which is negatively regulated by cytokinin [62, 63]. Moreover, down-regulated genes were also associated with steroid hormone biosynthesis, amino acid metabolism (arginine, proline, alanine, aspartate and glutamate), pyruvate metabolism, purine metabolism, endocytosis, the citrate cycle (TCA cycle), calcium signaling pathways and the metabolism of xenobiotics by cytochrome P450. Conversely, genes up-regulated by BA treatment in Clusters II, V and VII were associated with the cell cycle, DNA replication, the proteasome, pyrimidine metabolism and steroid hormone biosynthesis. Knowledge of the differential expression of transcripts encoding proteins involved in these pathways upon BA treatment will provide important clues regarding the mechanism of flower development responses to cytokinin in *Jatropha*.

**Table 4:** **KEGG pathway enrichment analysis of differentially expressed transcripts in eight clusters (p <0.05)**

Cluster	KEGG pathway	p-value
I	Apoptosis	0.002
	Steroid hormone biosynthesis	0.021
	Arginine and proline metabolism	0.029
	Alanine, aspartate and glutamate metabolism	0.03
II	Cell cycle	0.059
	DNA replication	0.097
III	--	--
IV	Pyruvate metabolism	0.017
	Apoptosis	0.018
V	Proteasome	0.0073
	Pyrimidine metabolism	0.038
VI	Apoptosis	0.0092
	Purine metabolism	0.014
	Endocytosis	0.019
	Pyruvate metabolism	0.021
	Citrate cycle (TCA cycle)	0.026
	Calcium signaling pathway	0.037
VII	DNA replication	0.041
	Steroid hormone biosynthesis	0.047
VIII	Metabolism of xenobiotics by cytochrome P450	0.027
	Apoptosis	0.036

### Genes involved in cytokinin biosynthesis, metabolism and signaling

The differentially expressed genes that were annotated to function in cytokinin biosynthesis, metabolism and signaling were further analyzed.

**Figure 4:**
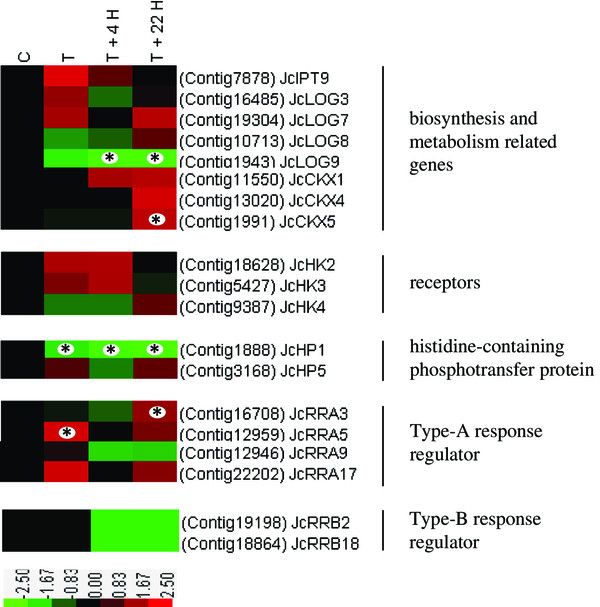
**Expression profiles of cytokinin biosynthesis- and metabolism-related genes and two-component elements following BA treatment.** Colors indicate the expression values scaled to the standard deviations and centered at the control intensity level (Z-score). Red indicates increased expression and green indicates decreased expression relative to the control condition. Gene expression at time points labeled with an asterisk on the map showed significantly differential expression between treated and control inflorescence meristems (p <0.05), whereas the remaining genes were considered to not respond to BA treatment (p >0.05).

Only one sequence in the transcriptome database, *isopentenyl transferase 9* (*IPT9*), was annotated as functioning in cytokinin biosynthesis. *Jatropha IPT9* (*JcIPT9*) was weakly expressed under both BA-treated and untreated conditions and was not responsive to BA treatment (Figure 4), which is consistent with previous results [52]. Four *Jatropha LONELY GUYs* (*JcLOG3*, *7*, *8*, and *9)*, which encode cytokinin-activating enzymes that function in the final step of bioactive cytokinin synthesis [64], were identified. *JcLOG3*, *7* and *8* also displayed weak expression across treated and untreated samples, whereas *JcLOG9* was down-regulated in response to BA (Figure 4). Three *Jatropha cytokinin oxidases/dehydrogenases* (*JcCKX1, 4,* and *5*) were present in the transcriptome database, and only *JcCKX5* was up-regulated by BA treatment (Figure 4). The stable induction of *CKX5* by cytokinin was confirmed in 9 of 13 microarray studies conducted by different labs [49].

**Figure 5:**
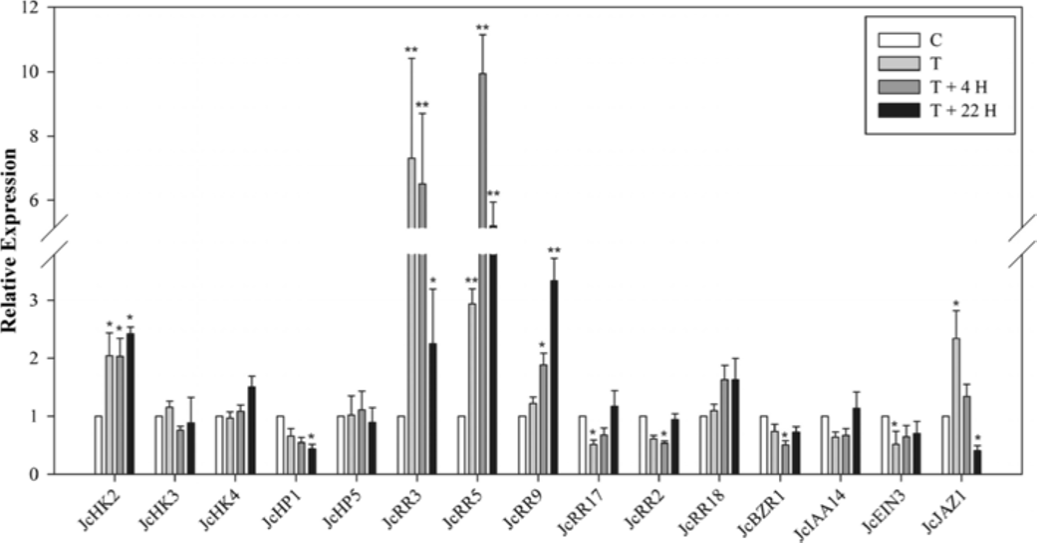
**Quantification of the expression of genes involved in cytokinin and other phytohormone signaling by qRT-PCR.** Results are shown as the relative expression of genes at different time points before and after BA treatment. Values are means ± standard deviations (n =3). *Statistically significant at the 5% level, **Statistically significant at the 1% level.

Previous genetic and molecular studies suggested that cytokinin signal transduction occurs through a two-component system [65–69]. Transcripts involved in two-component systems were identified in *Jatropha*, and their expression patterns are shown in Figure 4. Three *Jatropha* orthologs of *histidine kinases* (*JcHK2*, *3* and *4*), which act as cytokinin sensors [70], were identified. *JcHK2* was slightly induced by BA treatment (Figure 4), the expression of which detected by qRT-PCR was significantly higher than control after BA treatment in this study (Figure 5). The other two receptors, *JcHK3* and *4*, showed no clear response to BA treatment revealed by both transcriptome and qRT-PCR analysis (Figures 4 and 5). The gene coding for the *Jatropha* ortholog of histidine phosphotransfer protein 1 (*JcHP1*) was significantly repressed upon BA treatment, whereas *JcHP5* was not differentially regulated under our experimental conditions (Figures 4 and 5). Neither *HP1* nor *HP5* responded to cytokinin treatment in *Arabidopsis* [52, 66, 71]. However, repression of tomato *HP1* expression after BA treatment was reported by Shi et al. [34], who performed a transcriptome analysis of cytokinin responses in tomato leaves. Four *Jatropha* orthologs of type-A *response regulator* (*JcRRAs*) and two type-B *RR* genes (*JcRRBs*) were identified in this study. *JcRRA3* and *JcRRA5* were up-regulated by BA treatment (Figures 4 and 5), and in previous studies, *RRA3* and *RRA5* were robustly induced by cytokinin in *Arabidopsis* [49, 52, 72]. However, the mRNA levels of *JcRRA9* and *JcRRA17* showed no significant differences between the BA-treated and control samples (Figure 4). In contrast to the 454 transcriptome data, the expression level of *JcRRA9* determined by qRT-PCR showed a significant increase at T +22 H compared to control (Figure 5). Moreover, the transcription levels of *JcRRB2* and *JcRRB18,* which are B-type *RRs*, were not significantly affected by cytokinin (Figures 4 and 5), which agreed with previous studies on the differential expression of B-type response regulators in response to cytokinins [66, 72].

### Cytokinin-regulated genes involved in the metabolism and signaling of other phytohormones

**Figure 6:**
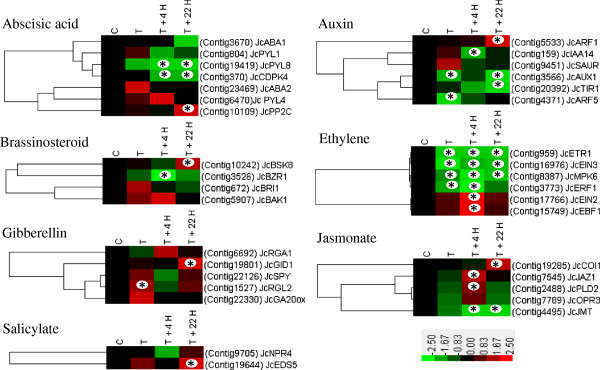
**Regulation of other phytohormone signaling genes following BA treatment revealed by DEGseq analysis of the 454 transcripts.** The colors indicate the expression values scaled to the standard deviations and centered at the control intensity level (Z-score). Red indicates increased expression and green indicates decreased expression relative to the control condition. Gene expression at time points labeled with an asterisk on the map showed differential expression between treated and control inflorescence meristems (p <0.05), whereas the remainder were considered to not respond to BA treatment (p >0.05).

Signaling pathways of phytohormones in plants are considered to be interconnected in a complex network [73]. Transcriptome studies indicated that cytokinin and auxin mutually regulate their signaling factors and/or their metabolism to control plant growth and development [48, 74]. In this study, the *Jatropha* ortholog of *AUXIN-RESISTANT1* (*JcAUX1*), which augments auxin’s chemiosmotic influx into cells [75], showed response to cytokinin with a decrease in transcript abundance (Figure 6). Additionally, the *Jatropha* ortholog of the gene encoding the F-box protein TRANSPORT INHIBITOR RESPONSE1 (*JcTIR1*), an auxin receptor [76], was also down-regulated by BA treatment at T +22 H (Figure 5). Similarly, the *Jatropha* ortholog of *INDOLEACETIC ACID-INDUCED PROTEIN 14* (*JcIAA14*), belonging to the *AUX/IAA* gene family, was also down-regulated (Figures 5 and 6). However, the *Jatropha* ortholog of another auxin-inducible gene that encodes small auxin up RNA (*JcSAUR*) [77, 78] was slightly but not significantly up-regulated by BA treatment (Figure 6). The *auxin response factor*s (*ARFs*), which bind to conserved DNA sequences called auxin-response elements (AuxREs) in the promoter regions of primary auxin response genes, act as transcriptional activators or repressors depending on the nature of their middle domain [79–81]. *Jatropha* orthologs of *ARF1* (*JcARF1*, transcriptional repressor) and *ARF5* (*JcARF5*, transcriptional activator) [82] were identified, and the mRNA level of *JcARF1* was increased whereas that of *JcARF5* was decreased (Figure 6). Moreover, *ARF5* was reported to repress *ARR7*/*15*, which negatively regulate cytokinin signaling in the shoot stem cell niche of *Arabidopsis* [82], indicating that cytokinin-regulated auxin signaling may be realized through the interaction between *ARR7/15* and *ARF5*. The expression changes of genes involved in auxin signal transduction upon BA treatment in *Jatropha* were more similar to those of tomato [34] than to those of *Arabidopsis* [52]. Taken together, these results indicate that intricate agonistic and antagonistic interactions exist between cytokinin and auxin in the development of inflorescence meristems in *Jatropha*. They also indicate differences may exist in the gene responses to cytokinin between *Jatropha* and the model plant *Arabidopsis*.

Following BA treatment, *Jatropha* orthologs of genes encoding a GA receptor, *GA INSENSITIVE DWARF1* (*JcGID1*), and a DELLA protein, *RGA LIKE 2* (*JcRGL2*), were up-regulated (Figure 6). A previous study of cytokinin response genes in *Arabidopsis* by Brenner et al. [54] also reported the induction of DELLA protein-encoding genes by cytokinin and reported that cytokinin treatment caused reduced expression of *GA20 oxidase*. However, in our study, BA did not cause changes in the transcript abundance of a *Jatropha* ortholog of *GA20 oxidase* (Figure 6). Earlier studies showed feedback regulation between GA content and *GID1* gene expression [83], and the amount of DELLA proteins was found to be inversely related to the amount of bioactive GA [84]. A *Jatropha* ortholog of another DELLA protein-encoding gene, *REPRESSOR OF ga1-1* (*JcRGA1*), was slightly up-regulated (Figure 6). The induction of these GA signaling genes by cytokinin in *Jatropha* inflorescence meristems suggests that cytokinin reduces GA activity by up-regulating suppressors of GA signaling genes and supports previous findings that GA and cytokinin exert antagonistic effects on many aspects of plant development [52, 85].

*Jatropha* orthologs of two ABA biosynthesis genes, *ABA DEFICIENT 1* (*JcABA1*) and *JcABA2*, were found to not be affected by BA treatment (Figure 6). However, we identified three *Jatropha* orthologs of ABA receptors, *PYR1-LIKE* (*PYL*) [86] *, JcPYL1, JcPYL4 and JcPYL8*, and one of them (*JcPYL8*) was significantly repressed by BA, whereas mRNA levels of *JcPYL1* and *JcPYL4* showed no clear response to BA treatment (Figure 6). In addition, a *Jatropha* ortholog of *protein phosphatase 2C* (*JcPP2C*), whose activity was inhibited by PYLs in response to ABA in *Arabidopsis* [86, 87], responded to BA with an increase of transcript abundance (Figure 6). Conversely, the expression of a *Jatropha* ortholog of *calcium-dependent protein kinase 4* (*JcCDPK4*), which is an important positive regulator in CDPK/calcium-mediated ABA signaling pathways at the whole-plant level in *Arabidopsis* [88], was down-regulated significantly by BA treatment (Figure 6). The BA responsiveness of *Jatropha* orthologs of genes involved in ABA signaling indicated an antagonistic interaction between cytokinin and ABA, which is in line with earlier studies revealing that cytokinins inhibit ABA production [89, 90].

Cytokinin also interacts with ethylene [91]. Decreases were identified in the transcript abundance of the *Jatropha* orthologs of *ethylene receptor 1* (*JcETR1*) and the gene encoding mitogen-activated protein kinase 6 (JcMPK6) (Figure 6), both of which are negative regulators of ethylene signaling [92, 93]. The *Jatropha* orthologs of two additional genes, *ethylene-responsive transcription factor 1* (*JcERF1*) and *ethylene insensitive protein 3* (*JcEIN3*), were also repressed by BA treatment (Figures 5 and 6). In *Arabidopsis*, *ethylene-insensitive protein 2* (*EIN2*) and *EIN3-binding F-box 1* (*EBF1*) are induced by ethylene [94, 95]. The *Jatropha* orthologs of both of these genes were up-regulated by BA treatment in the present study (Figure 6), indicating that positive regulators of the ethylene signaling pathway were induced by BA treatment. The expression profiles of these genes suggested that cytokinin may act partially by influencing ethylene metabolism and signaling genes [96].

Four *Jatropha* orthologs of genes involved in brassinosteroid (BR) signaling were identified. The expression levels of two of them, *BRASSINOSTEROID-INSENSITIVE 1* (*JcBRI1*) and *BRI1-ASSOCIATED RECEPTOR KINASE 1* (*JcBAK1*), which work together as a receptor kinase pair initiating the signal transduction cascade [97, 98], were not significantly altered by BA treatment (Figure 6). The *Jatropha* ortholog of *BRASSINOSTEROID-SIGNALING KINASE 8* (*JcBSK8*), which is in a small family of kinases that activate BR signaling downstream of *BRI* [99], was induced by BA (Figure 6). Furthermore, BA treatment inhibited the expression of the *Jatropha* ortholog of *BRASSINAZOLE RESISTANT 1* (*BZR1*) (Figures 5 and 6), which is a transcriptional repressor with dual roles in BR homeostasis and growth responses [100]. These findings indicate positive crosstalk between the cytokinin and BR signaling pathways in *Jatropha*.

Genes encoding the jasmonate ZIM-domain (JAZ) proteins, which are key regulators of jasmonate signaling [101], have been reported to repress transcription of jasmonate-responsive genes [102]. The transcription level of the *Jatropha* ortholog of *JAZ1* (*JcJAZ1*) increased upon BA treatment (Figures 5 and 6). The *Jatropha* ortholog of *ENHANCED DISEASE SUSCEPTIBILITY 5* (*JcEDS5*) was up-regulated, whereas the *Jatropha* ortholog of *NONEXPRESSOR OF PATHOGENESIS-RELATED GENES 4* (*JcNPR4*) showed no response to BA treatment (Figure 6). Both of *EDS5* and *NPR4* are involved in salicylate signaling [103]. These results imply that jasmonate and salicylate signaling may involve crosstalk with cytokinin in *Jatropha*.

### Genes involved in flower development

**Figure 7:**
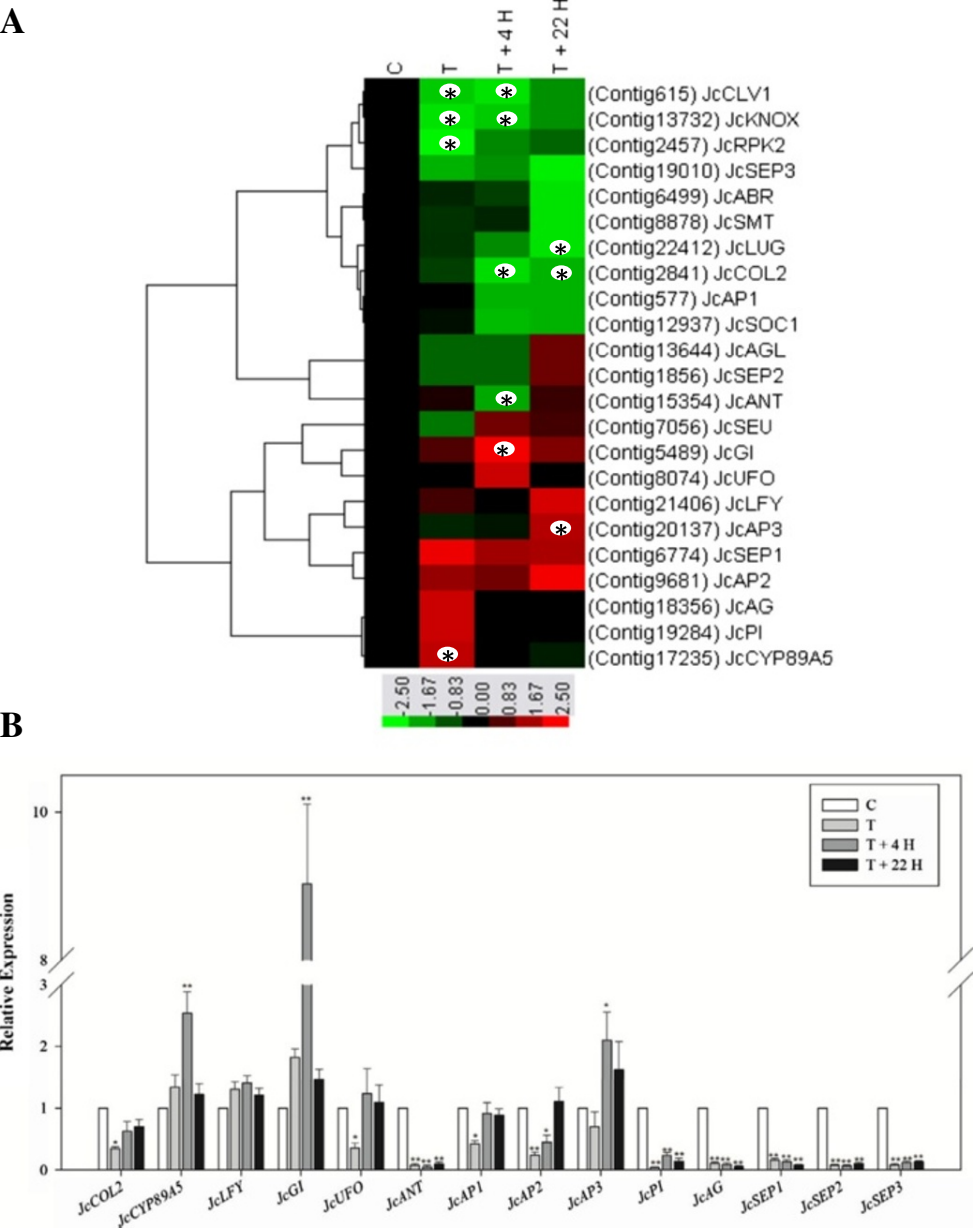
**Expression profiles of genes involved in flower development. (A)** Expression profiles of genes involved in flower development obtained by the DEGseq analysis of the 454 transcripts. The colors indicate the expression values scaled to the standard deviations and are centered at the control intensity level (Z-score). Red indicates increased expression and green indicates decreased expression relative to the control condition. Gene expression at time points labeled with an asterisk on the map showed differential expression between the treated and control inflorescence meristems (p <0.05), whereas the remaining genes were considered to not respond to BA treatment (p >0.05). **(B)** Quantification of gene expression by qRT-PCR. Results are shown as the relative expression of genes at different time points before and after BA treatment. Values are means ± standard deviations (n =3). **Statistically significant at the 1% level.

BA treatment profoundly affected *Jatropha* flower development (Figure 1). The expression profiles of *Jatropha* orthologs of 23 flowering-related genes identified in *Jatropha* inflorescence meristems are presented in Figure 7A. And the relative expression of 14 of these genes were further confirmed by qRT-PCR (Figure 7B). The mRNA level of the *Jatropha* ortholog of *CONSTANS-LIKE2* (*JcCOL2*), which contains a CCT (CONSTANS, CONSTANS-LIKE, and TIMING OF CAB1) domain and is related to flowering in response to the photoperiod [104], was significantly decreased by BA treatment (Figure 7A,B). In contrast, expression of tomato *COL* (Solyc07g006630) in leaves and *Arabidopsis COL* (At5g15850) in *CKX1*-overexpressing plants was induced upon treatment with cytokinin [34, 52]. The transcription level of the *Jatropha* ortholog of *CYP89A5*, a member of the *P450* gene family, increased after BA treatment (Figure 7). Genes in the P450 gene family in *Arabidopsis* and tomato were also induced by cytokinin [34, 48, 52], suggesting that *CYP89A5* may be involved in *Jatropha* inflorescence development in response to cytokinin. The transcript annotated to encode the *Jatropha* ortholog of *GIGANTEA* (*JcGI*), a circadian clock-controlled gene that regulates photoperiodic flowering in *Arabidopsis* [105, 106], was significantly induced at T +4 H (Figure 7A,B). Li et al. [13] reported that *LEAFY* (*LFY*), a floral meristem identity gene, was up-regulated by the accumulation of endogenous cytokinin in *Arabidopsis*. However, in our study, the *Jatropha* ortholog of *LFY* (*JcLFY*) showed no response to BA treatment (Figure 7A,B). The *Jatropha* ortholog of *UNUSUAL FLORAL ORGANS* (*JcUFO*), which encodes an F-box protein required for normal patterning and growth in the floral meristem in *Arabidopsis* [107], was down-regulated by BA (Figure 7B). *AINTEGUMENTA* (*ANT*) has been proposed to act downstream of auxin and involve in floral initiation, growth and patterning in *Arabidopsis* [108, 109]. After treatment with BA, *Jatropha* ortholog of *ANT* (*JcANT*) was profoundly down-regulated (Figure 7A,B). In *Arabidopsis*, *ant* flowers have fewer and smaller floral organs and possess ovules lacking integuments and a functional embryo sac [110, 111]. However, with decreased mRNA level of *JcANT*, flowers were not shown abnormal phenotypes in *Jatropha* after exposing to BA. Furthermore, *Jatropha* orthologs of other flowering-related genes such as *CLAVATA1* (*JcCLV1*), *KNOTTED-LIKE HOMEOBOX* (*JcKNOX*), *transcriptional corepressor LEUNIG* (*JcLUG*), and *serine/threonine protein kinase ABRUPTUS* (*JcABR*) were also down-regulated (Figure 7A).

The ABCE model was formulated from the analysis of floral homeotic mutants with organ identity defects in two adjacent whorls of the flower, and similar classes of mutants were described in both *Arabidopsis* and *Antirrhinum*, suggesting that the regulation of organ identity was highly conserved in evolution [112–114]. In this study, *Jatropha* orthologs of A-function genes *APETALA 1,2* (*JcAP1,2*), B-function gene *PISTILLATA* (*JcPI*), C-function gene *AGAMOUS* (*JcAG*) and E-function genes *SEPALLATA1,2,3* (*JcSEP1,2,3*) were significantly repressed, with an exception of one B-function gene *JcAP3* that was shown to be up-regulated by BA treatment (Figure 7A,B). Along with the altered expression of these ABCE model genes, bisexual and asexual flowers were induced, but no abnormal floral organ development was found in *Jatropha* treated with BA (Additional file 1: Figure S1, [10]). In *Arabidopsis*, exogenous BA application resulted in abnormal flowers that resemble the phenotypes of mutants, *clv1*, *ap1*, *ap2*, and *ap3* [22, 115]. However, the increased mRNA levels of *AP1*, *PI* and *AG* in transgenic *Arabidopsis*, which resulted from the expression of *AtIPT4* under control of *AP1*, also caused abnormal flower and floral organ development [116]. The different phenotypic changes in *Arabidopsis* [22, 115] and *Jatropha* occurring upon BA treatment (Additional file 1: Figure S1, [10]) likely resulted from the differential responses of flowering-related genes as revealed in this study and previous studies on *Arabidopsis* [12]. It is noteworthy that the relative expression of several genes involved in flower development were found to be significantly reduced after BA treatment by qRT-PCR analysis (Figure 7B), whereas their expression did not show significant difference compared to the control in transcriptome sequencing analysis (Figure 7A). This discrepancy probably resulted from the low background expression of these genes and/or the insufficient transcriptome sequencing depth. Normalizing the cDNA library and enhancing the depth of transcriptome sequencing may help to solve this problem.

### Cytokinin-regulated genes involved in cell division

**Figure 8:**
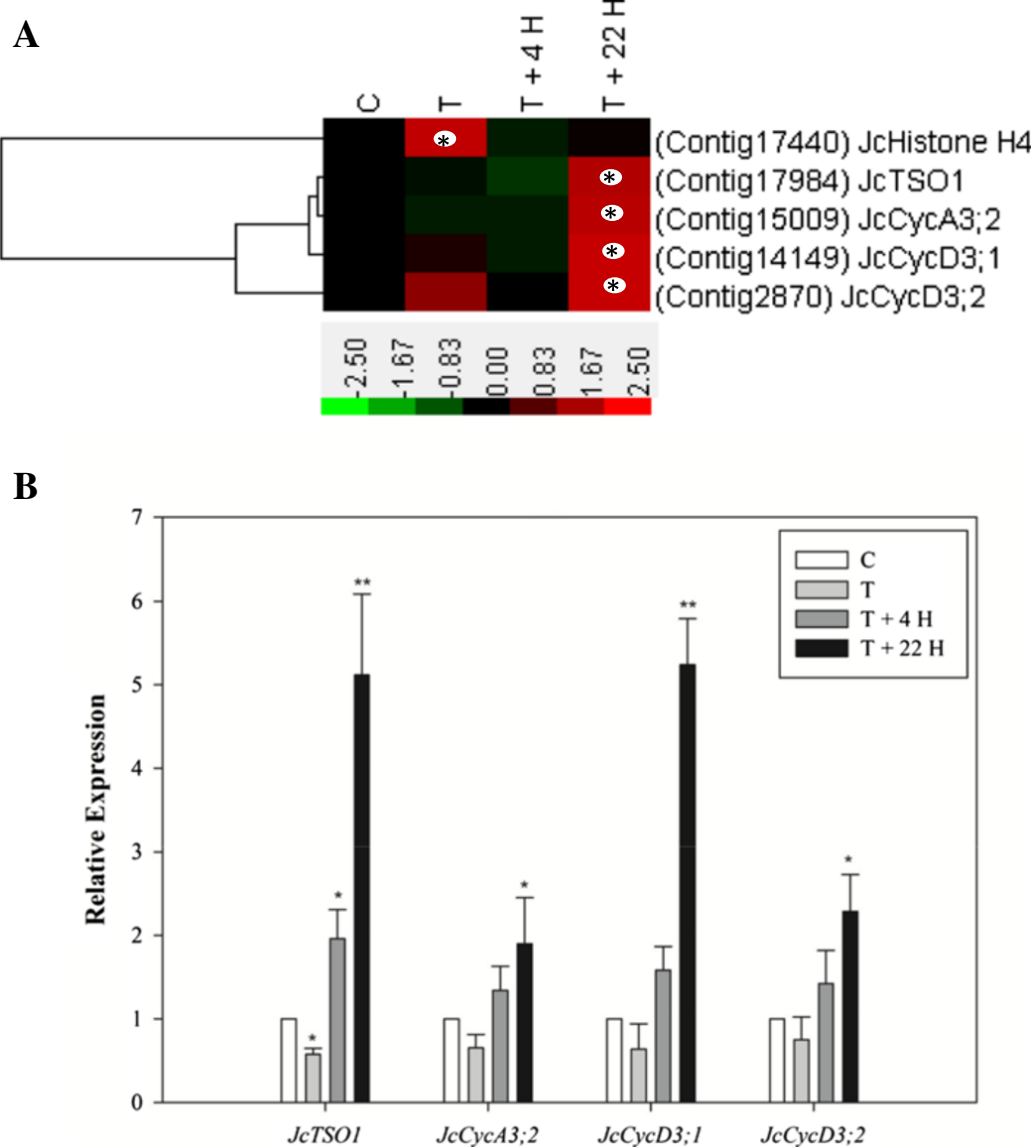
**Expression profiles of genes involved in cell division. (A)** Expression profiles of genes involved in cell division obtained by DEGseq analysis of the 454 transcripts. The colors indicate the expression values scaled to the standard deviations and are centered at the control intensity level (Z-score). Red indicates increased expression and green indicates decreased expression relative to the control condition. Gene expression at time points labeled with an asterisk on the map showed differential expression between treated and control inflorescences (p <0.05). **(B)** Quantification of gene expression by qRT-PCR. Results are shown as the relative expression of genes at different time points before and after BA treatment. Values are means ± standard deviations (n =3). *Statistically significant at the 5% level, **Statistically significant at the 1% level.

It was reported decades ago that cytokinin promotes cell division [117]. More recent work found that an elevated level of endogenous cytokinin in *Arabidopsis*, achieved by overexpression of *ATP/ADP isopentenyltransferase 4* (*AtIPT4*) [13] or loss-of-function of *CKX*, promoted cell division of the inflorescence meristem and the flower meristem [118–120]. In this study, expression of the S phase marker *histone H4* [64, 121] was found to be significantly higher in BA-treated (2 h) samples than in controls (Figure 8A). We identified four additional genes related to cell division in the database. Two *Jatropha* orthologs (Contig14149 and Contig2870) of *cyclin D3* (*JcCycD3;1* and *JcCycD3;2*), a D-type plant cyclin gene that plays an important role in the G1-to-S phase transition [121], were significantly up-regulated by BA treatment (Figure 8A,B). In *Arabidopsis*, cytokinin activates cell division through induction of *CycD3* [121–123]. In addition, the *Jatropha* ortholog of *cyclin A* (*JcCycA3;2*), an A-type plant cyclin gene that was reported to be associated with cell division [124, 125], was also induced following BA treatment (Figure 8A,B). *Arabidopsis TSO1* (*AtTSO1*) encodes a protein with conserved CXC domains known to bind DNA that is homologous to animal proteins that function in chromatin complexes [126, 127]. *AtTSO1* is involved in cell division during the development of the inflorescence meristem [128, 129]. Moreover, mutant alleles of *AtTSO1* that cause defects in flower and ovule development were identified [130, 131]. Subsequently, Andersen et al. [127] reported that mutations in the *AtTSO1* gene disrupt the development of both male and female reproductive tissues. *JcTSO1*, a *Jatropha* ortholog of *AtTSO1*, was highly expressed at T +22 H in *Jatropha* inflorescence meristems (Figure 8A,B). Based on this finding, we suggest that *JcTSO1*, together with other BA up-regulated and cell division associated genes (Figure 8), may be involved not only in promoting cell division in inflorescence meristems that resulted in increased total flower number per inflorescence after BA treatment (Figure 1, Additional file 1: Figure S1), but also in flower organ development that resulted in induced bisexual flowers upon BA treatment (Additional file 1: Figure S1, [10]).

## Conclusions

In this study, a time-course experiment was conducted to characterize activated and repressed genes in the inflorescence meristems of *Jatropha* following cytokinin treatment using transcriptome sequencing by a 454 GS FLX Titanium instrument. This approach produced 703,755 high-quality reads ranging from 100 bp to 790 bp, with an average length of 364 bp (Table 1). Up-regulated and down-regulated annotated and novel genes were identified (Figure 3, Additional file 3: Table S2), resulting in an unprecedented view of the regulatory activities of cytokinin in *Jatropha* inflorescence meristems.

Abundant physiological data suggest that plant hormones interact with each other to regulate various aspects of development [132–135]. In this study, the cytokinin signaling pathway was found to crosstalk with other signals, mainly through pathways converging on or through transcriptional factors or other signaling components. For example, interactions with GA can occur through induction of the negative regulators *JcGID1* and *JcRGL1*. The expression profiles of genes involved in other hormone signaling pathways indicated that: 1) there are multiple agonistic and antagonistic effects between cytokinin and auxin; 2) cytokinin may reduce plant responsiveness to GA and ABA by repressing GA and ABA metabolism- and signaling-related genes and 3) cytokinin may act synergistically with ethylene and BR in controlling *Jatropha* inflorescence meristem development.

**Figure 9:**
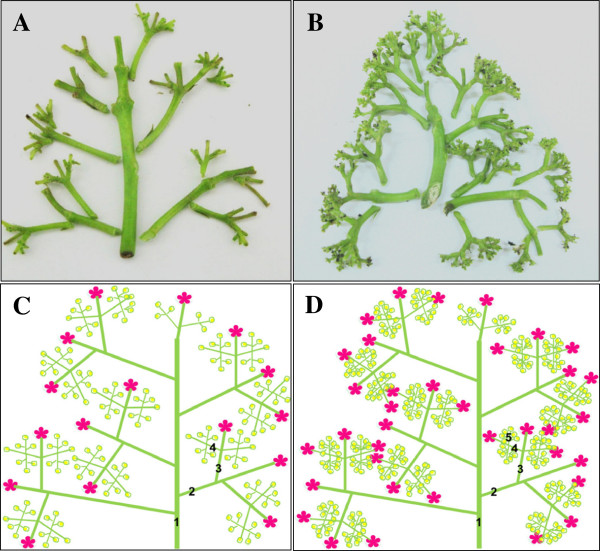
**BA treatment enhanced inflorescence branching of** ***Jatropha*** **.** **(A)** Branching of control inflorescence. **(B)** Branching of BA-treated inflorescence. **(C)** Diagram of branching of control inflorescence. **(D)** Diagram of branching of BA-treated inflorescence. To clearly show the pattern of inflorescence branching, the flowers in **(A)** and (B) were removed. The numbers (1–5) on the branches in **(C)** and **(D)** represent different orders of branching.  represents a female flower;  represents a male flower.

Exogenous BA treatments resulted in different phenotypic changes between *Jatropha* (Additional file 1: Figure S1, [10]) and *Arabidopsis* [22, 115]. And the response of ABCE model genes to CK in *Jatropha* (Figure 7) was also different from that of *Arabidopsis* [116]. Our analysis also indicates the increased total flower number per inflorescence of *Jatropha* after BA treatment (Figure 1, Additional file 1: Figure S1) resulted from the enhanced inflorescence branching, which was demonstrated by the occurrence of the fifth order branching in BA-treated inflorescences, whereas only the fourth order branching was found in control inflorescences (Figure 9, Additional file 4: Table S3). Therefore the up-regulation of genes related to cell division, especially *JcCycD3* and *JcTSO1*, likely contributed to the increased flower number per inflorescence after BA treatment in *Jatropha*. BA-induced expression of *JcCycD3* and *JcTSO1* caused production of more cells in the inflorescence meristems, which leads to an increase in size of inflorescence meristems and therefore an enhanced inflorescence branching, and results in more flowers. This hypothesis is supported by a recent study showing that the elevated CK levels in the reproductive SAM of rice resulted in increased meristem activity, enhanced panicle branching, and a consequent increase of grain number [136]. The functions of *JcCycD3* and *JcTSO1* will be further analyzed using transgenic *Jatropha* plants.

Like most of the published reports of RNA-seq data [31, 32, 137, 138], due to the high cost of 454 sequencing at the commencement of this study, only a single biological replicate was included for transcriptome analysis, which prevented proper statistical testing on identification of differential expressed genes. Nonetheless, different transcript abundance of the annotated genes in control and BA-treated inflorescences revealed by 454 sequencing in this study provide a valuable data source for selecting putative BA-regulated *Jatropha* genes for further verification by qRT-PCR with sufficient biological replicates. In this study, we did each qRT-PCR experiment with three biological and three technical replicates per biological replicate. Our results, as shown in Figures 4, 5, 6, 7 and 8, indicated a high correlation of expression levels between 454 sequencing data and qRT-PCR, but the congruence of statistically significant differential expression revealed by the two approaches was low. This observation is consistent with previous work showing lack of congruence between 454 sequencing and qRT-PCR results regarding genes predicted as significantly differential expression [139].

To our knowledge, this is the first report on the transcriptional regulation and identification of genes that are differentially expressed in the inflorescence meristems of *Jatropha* exposed to cytokinin. We have identified a set of cytokinin-regulated genes in *Jatropha* inflorescence meristems through expression profiling. Some of them correspond to previously identified genes in *Arabidopsis*, and others show different expression patterns from their *Arabidopsis* orthologs. Further analyses of these genes with different expression patterns are needed to elucidate their roles in the cytokinin responses of *Jatropha* inflorescence meristems. Although transcriptional analysis is only an initial step and does not identify functional relevance as it may or may not relate to changes in the level and/or activity of the corresponding proteins, the potential cytokinin-responsive transcripts identified in this study will provide a good starting point for investigations into the molecular mechanisms of *Jatropha* responses to cytokinin.

## Methods

### Plant material and BA treatment

Cuttings from one *Jatropha* plant were propagated into individual plants and used as experimental plants. BA treatment was performed when the plants were undergoing the flowering stage in the following year. In total, 230 inflorescence meristems with a diameter of approximately 0.5 cm were selected as experiment subjects. First, each meristem was wrapped around by a piece of absorbent cotton weighing 10 mg. Then, 200 μl of a 1 mM BA solution containing 0.05% Tween-20 was applied to the cotton using a pipette.

Before BA treatment, 50 meristems were collected as control. Two hours after treatment, all cotton pieces wrapped around the inflorescence meristems were removed, and 50 meristems were sampled, which were identified as T (BA treatment for 2 hours). Fifty meristems were sampled at 4 and 22 hours post BA treatment, which were identified as T +4 H and T +22 H, respectively. The remaining 30 meristems were kept for phenotypic analysis. The fifty inflorescence meristems sampled at each time point, which were pooled together as one sample for 454 sequencing, were immediately frozen in liquid nitrogen and stored at −80°C.

To investigate the effect of cytokinin on branching of inflorescence, BA working solution (0.5 mM) with 0.05% (v/v) Tween-20 was sprayed onto each inflorescence meristem (about 0.5 cm in diameter) and the surrounding leaves using a hand sprayer. Control inflorescence meristems were sprayed with distilled water containing 0.05% (v/v) Tween-20. Spraying was conducted once. Thirty inflorescence meristems were used for each treatment.

### RNA extraction, cDNA synthesis and 454 sequencing

Each frozen sample was ground in a mortar with liquid nitrogen, and total RNA was isolated using TRIzol reagent (Invitrogen Corp., Carlsbad, CA) following the standard protocol. An Agilent 2100 instrument was used to check the RNA quality (RIN >0.7), and a NanoDrop spectrophotometer (ND-2000C, Thermo Fisher Scientific, USA) was used to quantify RNA concentration. Messenger RNA was further purified using a MicroPoly(A) Purist Kit (Ambion) according to the protocol. Double-stranded cDNA was synthesized from mRNA according to Ng's full-length cDNA synthesis protocol [140] with some modifications [141] and then fragmented to 300–800 bp. The prepared cDNAs were transformed into single-stranded template DNA (sstDNA) libraries using the GS DNA Library Preparation kit (Roche Applied Science). sstDNA libraries were clonally amplified in a bead-immobilized form using the GS emPCR kit (Roche Applied Science) and sequenced on the 454 Genome Sequencer FLX instrument.

### Sequence assembly and annotation

The 454 transcriptome sequencing reads were first handled by a 454 GS FLX system, which cut off the adapter and low-quality bases. The reads were then filtered by an in-house-developed program to remove low-quality reads. The qualified reads were then assembled by CAP3 using the default parameters [47]. Open reading frames were identified using an in-house-developed program based on ‘GetORF’ from EMBOSS [142], and the annotation was performed through BLAST searches against the Swiss-Prot and GenBank databases with an E-value cutoff of 1E-3. Gene ontology analysis was performed using GoPipe through BLASTP against the Swiss-Prot and TrEMBL databases using an E-value cutoff of 1E-3 [143]. The metabolic pathway was constructed based on the KEGG database by the BBH (bi-directional best hit) method [144].

### Analysis of differentially expressed genes

To estimate gene expression, the read number for each gene was first transformed into RPKM (reads per kilobase per million reads) [145], and differentially expressed genes were identified by the DEGseq (identifying differentially expressed genes from gene expression data) package using the method MARS (MA-plot-based method with random sampling model) [146]. We use p-value <0.05 and the absolute value of log2Ratio >1 as the threshold to judge the significance of contig expression difference.

We perform BLASTX (NT query to AA database) in TAIR WU Blast against TAIR10 *Arabidopsis* proteins using NO filters to identify the most highly similar genes involved in plant hormone signaling, flower development and cell division (Additional file 5: Table S4). The members of the two-component signaling pathway in *Jatropha* was named following the nomenclature reported by Heyl et al. [147].

We used Z score transformation [148] to perform the normalization in Figure 3, which are calculated by subtracting the mean RPKM (reads per kilobase per million reads), and dividing that result by the standard deviation (SD) of four time points for each gene, according to the formula:


Where G is any gene from the transcriptome database.

We used Z score transformation with some modifications to perform the normalization in heat maps of Figures 4, 6, 7 and 8, which are calculated by subtracting the control RPKM, and dividing that result by the standard deviation (SD) of four time points for each gene, according to the formula:


Where G is any gene from the transcriptome database and RPKM_control_ is the RPKM of control.

We identified enriched GO terms and pathways for the differentially expressed genes using DAVID (the Database for Annotation, Visualization and Integrated Discovery). DAVID is a web-based bioinformatics application that systematically identifies enrichment for biological annotations based on large gene lists derived from high-throughput genomic experiments [50, 51].

### Quantitative real-time PCR (qRT-PCR) confirmation

The expression profiles of 15 genes involved in plant hormone signaling, 14 genes associated with flowering and 4 genes involved in cell division were investigated by qRT-PCR to confirm the transcriptome data. The RNA samples used for qRT-PCR were isolated from tissues collected from the experiment used for constructing the 454 libraries, and from inflorescence meristems collected in a replicate experiment. cDNA for each sample was synthesized from total RNA using the PrimeScript RT reagent Kit with gDNA Eraser (Perfect Real Time) (Takara, Japan) according to the instructions. The primers used for qRT-PCR are listed in Additional file 6: Table S5. The PCR products were sent to BGI (Shenzhen, China) for sequencing using specific primers for sequence confirmation. Relative gene expression levels were detected using the SYBR Premix Ex Taq II (Tli RNaseH Plus) (Takara, Japan) according to the manufacturer’s instructions on a LightCycler 480 II (Roche, USA) instrument. The relative expression of each gene was calculated using the 2^-ΔΔC^_T_ method [149]. All quantitative PCR experiments were repeated with three biological and three technical replicates per biological replicate.

### Accession numbers

All of the sequences of the unigenes greater than 200-bp in length obtained from the transcriptome sequencing of inflorescence meristems in *Jatropha* have been deposited in the Transcriptome Shotgun Assembly (TSA) database, http://www.ncbi.nlm.nih.gov/bioproject/265802 (Accession: PRJNA265802;ID: 265802). Unigenes less than 200-bp in length were listed in Additional file 7: Table S6.

## Electronic supplementary material

Additional file 1: Figure S1: Effects of BA on flower development and fruiting of *Jatropha*. **(A)** Effects of BA treatment on the flower number of various sexes per inflorescence and fruit number per infructescence. **(B)** Effects of BA treatment on the percentage of flowers of various sexes. The values are means ± standard deviations (n =30 inflorescences). *Statistically significant at the 5% level, **Statistically significant at the 1% level. (PDF 91 KB)

Additional file 2: Table S1: Top-hit species distribution of BLAST matches of *Jatropha* unigenes with a cutoff of 1e-3. (DOCX 17 KB)

Additional file 3: Table S2: Clustering of differentially expressed genes in the transcriptomes of control and BA-treated inflorescence of *Jatropha* (p <0.05). (XLSX 1 MB)

Additional file 4: Table S3: BA treatment enhanced inflorescence branching of *Jatropha*. (DOCX 26 KB)

Additional file 5: Table S4: BLASTX results of genes involved in plant hormone signaling, flower development and cell division against TAIR. (DOCX 30 KB)

Additional file 6: Table S5: Primer sequences and PCR amplicon lengths of the selected genes. (DOCX 18 KB)

Additional file 7: Table S6: Sequences of unigenes less than 200-bp in length obtained from the transcriptome sequencing of inflorescence meristems in *Jatropha.* (ZIP 12 KB)
