# Supplementary figures and images for: Transcriptome of the inflorescence meristems of the biofuel plant Jatropha curcas treated with cytokinin
Source: BMC Genomics. 2014 Nov 17;15(1):974. doi: 10.1186/1471-2164-15-974 (PMC4246439; doi:10.1186/1471-2164-15-974)

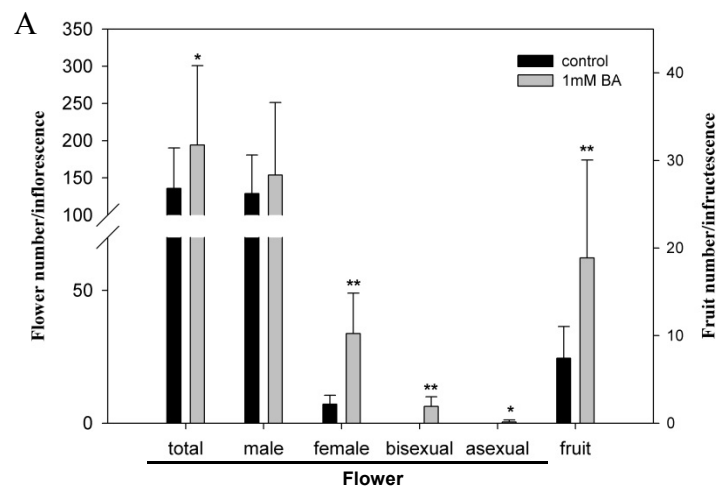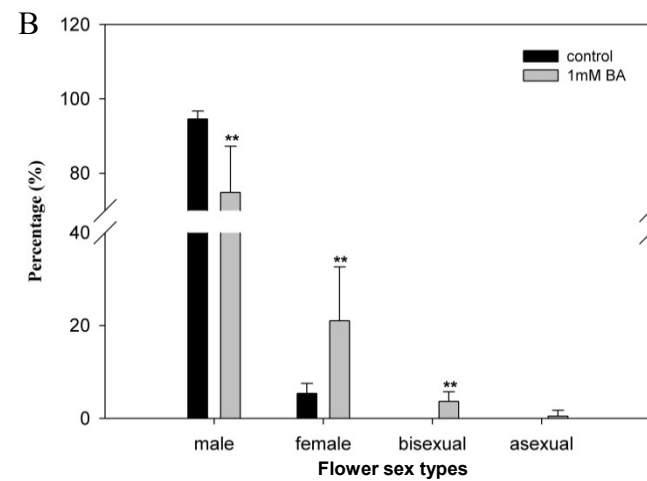

Supplement: Supplementary file 1 — Additional file 1: Figure S1: Effects of BA on flower development and fruiting of Jatropha. (A) Effects of BA treatment on the flower number of various sexes per inflorescence and fruit number per infructescence. (B) Effects of BA treatment on the percentage of flowers of various sexes. The values are means ± standard deviations (n =30 inflorescences). *Statistically significant at the 5% level, **Statistically significant at the 1% level. (PDF 91 KB) [file 12864_2014_6670_MOESM1_ESM.pdf]
