# Supplementary material for: Transcriptome of the inflorescence meristems of the biofuel plant Jatropha curcas treated with cytokinin
Source: BMC Genomics. 2014 Nov 17;15(1):974. doi: 10.1186/1471-2164-15-974 (PMC4246439; doi:10.1186/1471-2164-15-974)
Supplement: Supplementary file 2 — Additional file 2: Table S1: Top-hit species distribution of BLAST matches of Jatropha unigenes with a cutoff of 1e-3. (DOCX 17 KB) [file 12864_2014_6670_MOESM2_ESM.docx]

Table S1 Top-hit species distribution of BLAST matches of Jatropha unigenes with a

cutoff of 1e-3.

| Species | Top hit number | | Total | Percentage (%) |
| --- | --- | --- | --- | --- |
|  | Contig | Sigleton |  |  |
| *Ricinus communis* | 11824 | 15317 | 27141 | 58.41 |
| *Populus trichocarpa* | 3522 | 5297 | 8819 | 18.98 |
| *Vitis vinifera* | 1385 | 2527 | 3912 | 8.42 |
| *Arabidopsis thaliana* | 85 | 291 | 376 | 0.81 |
| *Glycine max* | 116 | 250 | 366 | 0.79 |
| *Zea mays* | 61 | 261 | 322 | 0.69 |
| *Sorghum bicolor* | 52 | 260 | 312 | 0.67 |
| *Arabidopsis lyrata* | 62 | 191 | 253 | 0.54 |
| *Oryza sativa* | 64 | 176 | 240 | 0.52 |
| other species | 1172 | 3551 | 4723 | 10.16 |
