# Supplementary material for: Transcriptome of the inflorescence meristems of the biofuel plant Jatropha curcas treated with cytokinin
Source: BMC Genomics. 2014 Nov 17;15(1):974. doi: 10.1186/1471-2164-15-974 (PMC4246439; doi:10.1186/1471-2164-15-974)
Supplement: Supplementary file 4 — Additional file 4: Table S3: BA treatment enhanced inflorescence branching of Jatropha. (DOCX 26 KB) [file 12864_2014_6670_MOESM4_ESM.docx]

Table S4 BLASTX results of genes involved in plant hormone signaling, flower development and cell division against TAIR.

| Gene name | Abbreviation | Contig | TAIR Blast Top Hit | |
| --- | --- | --- | --- | --- |
|  |  |  | Locus | Description |
| *Jatropha ABA DEFICIENT 1* | *JcABA1* | Contig3670 | AT5G67030 | Encodes a single copy zeaxanthin epoxidase gene that functions in first step of the biosynthesis of the abiotic stress hormone abscisic acid (ABA) |
| *Jatropha ABA DEFICIENT 2* | *JcABA2* | Contig23469 | AT1G52340 | Encodes a cytosolic short-chain dehydrogenase/reductase involved in the conversion of xanthoxin to ABA-aldehyde during ABA biosynthesis. |
| *Jatropha ABRUPTUS* | *JcABR* | Contig6499 | AT2G34650 | Encodes a protein serine/threonine kinase that may act as a positive regulator of cellular auxin efflux, as a binary switch for PIN polarity, and as a negative regulator of auxin signaling. |
| *Jatropha AGAMOUS* | *JcAG* | Contig13323 | AT4G18960 | Floral homeotic gene encoding a MADS domain transcription factor. |
| *Jatropha AGAMOUS-LIKE* | *JcAGL* | Contig13644 | AT3G58780 | Control dehiscence zone differentiation and promote the lignification of adjacent cells. |
| *Jatropha AINTEGUMENTA* | *JcANT* | Contig15354 | AT4G37750 | ANT is required for control of cell proliferation and encodes a putative transcriptional regulator similar to AP2. |
| *Jatropha APETALA 1* | *JcAP1* | Contig3340 | AT1G69120 | Floral homeotic gene encoding a MADS domain protein homologous to SRF transcription factors. |
| *Jatropha APETALA 3* | *JcAP3* | Contig17526 | AT3G54340 | Floral homeotic gene encoding a MADS domain protein homologous to SRF transcription factors. Specifies petal and stamen identities. |
| *Jatropha auxin response factor 1* | *JcARF1* | Contig5533 | AT1G59750 | Encodes a member of the auxin response factor family. ARFs bind to the *cis* element 5'-TGTCTC-3' ARFs mediate changes in gene expression in response to auxin. |
| *Jatropha auxin response factor 5* | *JcARF5* | Contig4371 | AT1G19850 | Encodes a transcription factor mediating embryo axis formation and vascular development. Similar toARF1 shown to bind to auxin responsive elements (AREs). |
| *Jatropha AUXIN-RESISTANT1* | *JcAUX1* | Contig3566 | AT2G38120 | Encodes an auxin influx transporter. AUX1 resides at the apical plasma membrane of protophloem cells and at highly dynamic subpopulations of Golgi apparatus. |
| *Jatropha BRI1-ASSOCIATED RECEPTOR KINASE 1* | *JcBAK1* | Contig5907 | AT4G33430 | Leu-rich receptor Serine/threonine protein kinase. Component of BR signaling that interacts with BRI1 in vitro and in vivo to form a heterodimer. |
| *Jatropha BRASSINOSTEROID*  *-INSENSITIVE 1* | *JcBRI1* | Contig672 | AT4G39400 | Encodes a plasma membrane localized leucine-rich repeat receptor kinase involved in brassinosteroid signal transduction. |
| *Jatropha BRASSINOSTEROID -SIGNALING KINASE 8* | *JcBSK8* | Contig10242 | AT5G41260 | Protein kinase protein with tetratricopeptide repeat domain; FUNCTIONS IN: binding, protein kinase activity, kinase activity, ATP binding. |
| *Jatropha BRASSINAZOLE RESISTANT 1* | *JcBZR1* | Contig3526 | AT1G75080 | Encodes a positive regulator of the brassinosteroid (BR) signaling pathway that mediates both downstream BR responses and negative feedback regulation of BR biosynthesis. |
| *Jatropha calcium-dependent*  *protein kinase 4* | *JcCDPK4* | Contig370 | AT4G09570 | Encodes a member of Calcium Dependent Protein Kinase (CDPK) gene family. Positive regulator of ABA signaling. |
| *Jatropha cytokinin oxidases/dehydrogenase 1* | *JcCKX1* | Contig11550 | AT2G41510 | Encodes a protein whose sequence is similar to cytokinin oxidase/dehydrogenase, which catalyzes the degradation of cytokinins. |
| *Jatropha cytokinin oxidases/dehydrogenase 4* | *JcCKX4* | Contig13020 | AT4G29740 | Encodes a protein whose sequence is similar to cytokinin oxidase/dehydrogenase, which catalyzes the degradation of cytokinins. |
| *Jatropha cytokinin oxidases/dehydrogenase 5* | *JcCKX5* | Contig1991 | AT1G75450 | Encodes a protein whose sequence is similar to cytokinin oxidase/dehydrogenase, which catalyzes the degradation of cytokinins. |
| *Jatropha CLAVATA1* | *JcCLV1* | Contig615 | AT5G65700 | Encodes a CLAVATA1-related receptor kinase-like protein required for both shoot and flower meristem function. |
| *Jatropha CORONATINE INSENSITIVE 1* | *JcCOI1* | Contig19285 | AT2G39940 | Encodes a protein containing Leu-rich repeats and a degenerate F-box motif. |
| *Jatropha CONSTANS-LIKE2* | *JcCOL2* | Contig2841 | AT3G02380 | Homologous to the flowering-time gene CONSTANS (CO) encoding zinc-finger proteins. |
| *Jatropha cyclin A3;2* | *JcCycA3;2* | Contig15009 | AT1G47210 | Cyclin-dependent protein kinase 3;2 (CycA3;2). |
| *Jatropha cyclin D3;1* | *JcCycD3;1* | Contig14149 | AT4G34160 | Encodes a cyclin D-type protein involved in the switch from cell proliferation to the final stages of differentiation. The gene is transcriptionally regulated by cytokinin and brassinosteroid. |
| *Jatropha cyclin D3;2* | *JcCycD3;2* | Contig2870 | AT5G67260 | Encode CycD3;2. Important for determining cell number in developing lateral organs. Mediating cytokinin effect in apical growth and development. |
| *Jatropha CYP89A5* | *JcCYP89A5* | Contig17235 | AT1G64950 | Cytochrome P450 superfamily protein. |
| *Jatropha EIN3-binding*  *F-box 1* | *JcEBF1* | Contig15749 | AT2G25490 | Encodes an F-box protein involved in the ubiquitin/proteasome-dependent proteolysis of EIN3. |
| *Jatropha ENHANCED*  *DISEASE SUSCEPTIBILITY 5* | *JcEDS5* | Contig19644 | AT4G39030 | Encodes an orphan multidrug and toxin extrusion transporter. Essential component of salicylic acid-dependent signaling for disease resistance. |
| *Jatropha ethylene-insensitive*  *protein 2* | *JcEIN2* | Contig17766 | AT5G03280 | Involved in ethylene signal transduction. Acts downstream of CTR1. Positively regulates ORE1 and negatively regulates mir164A, B, C to regulate leaf senescence. |
| *Jatropha ethylene*  *insensitive protein 3* | *JcEIN3* | Contig16976 | AT3G20770 | Encodes EIN3, a nuclear transcription factor that initiates downstream transcriptional cascades for ethylene responses. |
| *Jatropha ethylene-responsive transcription factor 1* | *JcERF1* | Contig3773 | AT4G17500 | Encodes a member of the ethylene response factor subfamily B-3 of ERF/AP2 transcription factor family (ATERF-1). |
| *Jatropha ethylene receptor 1* | *JcETR1* | Contig959 | AT1G66340 | Similar to prokaryote sensory transduction proteins. Contains a histidine kinase and a response regulator domain. |
| *Jatropha GA20 oxidase* | *JcGA20ox* | Contig22330 | AT5G51810 | Encodes gibberellin 20-oxidase. Involved in gibberellin biosynthesis. |
| *Jatropha GA*  *INSENSITIVE DWARF1* | *JcGID1* | Contig19801 | AT5G27320 | Encodes a gibberellin (GA) receptor ortholog of the rice GA receptor gene (OsGID1). Interacts with DELLA proteins in vivo in the presence of GA4. |
| *Jatropha GIGANTEA* | *JcGI* | Contig5489 | AT1G22770 | GI promotes flowering under long days in a circadian clock-controlled flowering pathway. |
| *Jatropha Histone H4* | *JcHistone H4* | Contig17440 | AT1G07660 | Histone superfamily protein. |
| *Jatropha histidine kinase 2* | *JcHK2* | Contig18628 | AT2G01830 | Histidine kinase: cytokinin-binding receptor that transduces cytokinin signals across the plasma membrane. |
| *Jatropha histidine kinase 3* | *JcHK3* | Contig5427 | AT1G27320 | Histidine kinase, a cytokinin receptor that controls cytokinin-mediated leaf longevity through a specific phosphorylation of the response regulator, ARR2. |
| *Jatropha histidine kinase 4* | *JcHK4* | Contig9387 | AT2G01830 | Histidine kinase: cytokinin-binding receptor that transduces cytokinin signals across the plasma membrane. |
| *Jatropha histidine*  *phosphotransfer protein 1* | *JcHP1* | Contig1888 | AT3G21510 | Encodes AHP1, function as redundant positive regulators of cytokinin signaling. |
| *Jatropha histidine*  *phosphotransfer protein 5* | *JcHP5* | Contig3168 | AT1G03430 | Encodes AHP5. |
| *Jatropha INDOLEACETIC ACID-INDUCED PROTEIN 14* | *JcIAA14* | Contig159 | AT4G14550 | A member of the Aux/IAA protein family. |
| *Jatropha isopentenyl*  *transferase 19* | *JcIPT9* | Contig7878 | AT5G20040 | Encodes tRNA isopentenyltransferase. |
| *Jatropha jasmonate*  *ZIM-domain (JAZ) protein 1* | *JcJAZ1* | Contig7545 | AT1G19180 | A nuclear-localized protein involved in jasmonate signaling. JAZ transcript levels rise in response to a jasmonate stimulus. |
| *Jatropha JASMONIC ACID CARBOXYL*  *METHYLTRANSFERASE* | *JcJMT* | Contig4495 | AT1G19640 | Encodes an enzyme that catalyzes the formation of methyljasmonate from jasmonic acid. Its expression is induced in response to wounding or methyljasmonate treatment. |
| *Jatropha KNOTTED-LIKE HOMEOBOX* | *JcKNOX* | Contig13732 | AT4G08150 | A member of class I knotted1-like homeobox gene family (together with KNAT2). Similar to the knotted1 (kn1) homeobox gene of maize. |
| *Jatropha LEAFY* | *JcLFY* | Contig21406 | AT5G61850 | Encodes transcriptional regulator that promotes the transition to flowering. Involved in floral meristem development. |
| *Jatropha LEUNIG* | *JcLUG* | Contig22412 | AT4G32551 | Regulates floral organ identity, gynoecium and ovule development. Negatively regulates AG. |
| *Jatropha LONELY GUY 3* | *JcLOG3* | Contig16485 | AT2G37210 | Encodes a protein of unknown function. It has been crystallized and shown to be structurally almost identical to the protein encoded by At5g11950. |
| *Jatropha LONELY GUY 7* | *JcLOG7* | Contig19304 | AT5G06300 | Putative lysine decarboxylase family protein. |
| *Jatropha LONELY GUY 8* | *JcLOG8* | Contig10713 | AT5G11950 | It has been crystallized and shown to be structurally almost identical to the protein encoded by At2G37210. |
| *Jatropha LONELY GUY 9* | *JcLOG9* | Contig1943 | AT1G50575 | Putative lysine decarboxylase family protein. |
| *Jatropha mitogen-activated protein kinase 6* | *JcMPK6* | Contig8387 | AT2G43790 | Encodes a MAP kinase induced by pathogens, ethylene biosynthesis, oxidative stress and osmotic stress. Also involved in ovule development. |
| *Jatropha PATHOGENESIS -RELATED GENES 4* | *JcNPR4* | Contig9705 | AT4G19660 | Encodes NPR4, ankyrin repeat BTB/POZ domain-containing protein. |
| *Jatropha OXOPHYTODIENOATE -REDUCTASE 3* | *JcOPR3* | Contig7789 | AT2G06050 | Encodes a 12-oxophytodienoate reductase that is required for jasmonate biosynthesis. |
| *Jatropha PHOSPHOLIPASED ALPHA 2* | *JcPLD2* | Contig2488 | AT1G52570 | Member of C2-PLD subfamily. |
| *Jatropha protein phosphatase 2C* | *JcPP2C* | Contig10109 | AT2G30020 | Encodes AP2C1. Belongs to the clade B of the PP2C-superfamily. Acts as a MAPK phosphatase that negatively regulates MPK4 and MPK6. |
| *Jatropha PYR1-LIKE 1* | *JcPYL1* | Contig804 | AT5G46790 | PYR/PYL/RCAR family proteins function as abscisic acid sensors. Mediate ABA-dependent regulation of protein phosphatase 2Cs ABI1 and ABI2. |
| *Jatropha PYR1-LIKE 4* | *JcPYL4* | Contig6470 | AT2G38310 | Abscisic acid sensors. |
| *Jatropha PYR1-LIKE 8* | *JcPYL8* | Contig19419 | AT5G53160 | Abscisic acid sensors. |
| *Jatropha REPRESSOR OF ga1-1* | *JcRGA1* | Contig6692 | AT2G01570 | Putative transcriptional regulator repressing the gibberellin response and integration of phytohormone signaling. |
| *Jatropha RGA LIKE 2* | *JcRGL2* | Contig1527 | AT3G03450 | Encodes a DELLA protein, a member of the GRAS superfamily of putative transcription factors. |
| *Jatropha RECEPTOR-LIKE PROTEIN KINASE 2* | *JcRPK2* | Contig2457 | AT3G02130 | Encodes a receptor-like kinase RPK2 (also known as TOADSTOOL 2/TOAD2). Functions as a regulator of meristem maintenance. |
| *Jatropha type-B*  *response regulator 2* | *JcRRB2* | Contig19198 | AT4G16110 | Type B response regulator. |
| *Jatropha type-A*  *response regulator 3* | *JcRRA3* | Contig16708 | AT1G59940 | Type A response regulator. |
| *Jatropha type-A*  *response regulator 5* | *JcRRA5* | Contig12959 | AT3G48100 | Type A response regulator. |
| *Jatropha type-A*  *response regulator 9* | *JcRRA9* | Contig12946 | AT3G57040 | Type A response regulator. |
| *Jatropha type-A*  *response regulator 17* | *JcRRA17* | Contig22202 | AT3G56380 | Type A response regulator. |
| *Jatropha type-B*  *response regulator 18* | *JcRRB18* | Contig18864 | AT5G58080 | Type B response regulator. |
| *Jatropha small*  *auxin up RNA* | *JcSAUR* | Contig9451 | AT4G38840 | SAUR-like auxin-responsive protein family. |
| *Jatropha SEPALLATA 1* | *JcSEP1* | Contig11701 | AT5G15800 | Encodes a MADS box transcription factor involved flower and ovule development. |
| *Jatropha SEPALLATA 2* | *JcSEP2* | Contig3088 | AT5G15800 | Encodes a MADS box transcription factor involved flower and ovule development. |
| *Jatropha SEPALLATA 3* | *JcSEP3* | Contig3464 | AT1G24260 | Encodes a MADS box transcription factor. |
| *Jatropha SEUSS* | *JcSEU* | Contig7056 | AT1G43850 | Encodes a transcriptional co-regulator of AG that functions with LEU to repress AG in the outer floral whorls. |
| *Jatropha SHOOT MERISTEMLESS* | *JcSMT* | Contig8878 | AT1G62360 | Class I knotted-like homeodomain protein that is required for shoot apical meristem (SAM) formation during embryogenesis and for SAM function throughout the lifetime of the plant. |
| *Jatropha SUPPRESSOR OF OVEREXPRESSION OF CO1* | *JcSOC1* | Contig12937 | AT2G45660 | Controls flowering and is required for CO to promote flowering. It acts downstream of FT. |
| *Jatropha SPINDLY* | *JcSPY* | Contig22126 | AT3G11540 | Encodes an N-acetyl glucosamine transferase that may glycosylate other molecules involved in GA signaling. |
| *Jatropha TRANSPORT INHIBITOR RESPONSE1* | *JcTIR1* | Contig20392 | AT3G62980 | Encodes an auxin receptor that mediates auxin-regulated transcription. |
| *Jatropha TSO1* | *JcTSO1* | Contig17984 | AT3G22780 | Putative DNA binding protein (TSO1). |
| *Jatropha UNUSUAL FLORAL ORGANS* | *JcUFO* | Contig8074 | AT1G30950 | Required for the proper identity of the floral meristem. Involved in establishing the whorled pattern of floral organs, in the control of specification of the floral meristem, and in the activation of APETALA3 and PISTILLATA. |
