# Supplementary material for: Transcriptome of the inflorescence meristems of the biofuel plant Jatropha curcas treated with cytokinin
Source: BMC Genomics. 2014 Nov 17;15(1):974. doi: 10.1186/1471-2164-15-974 (PMC4246439; doi:10.1186/1471-2164-15-974)
Supplement: Supplementary file 5 — Additional file 5: Table S4: BLASTX results of genes involved in plant hormone signaling, flower development and cell division against TAIR. (DOCX 30 KB) [file 12864_2014_6670_MOESM5_ESM.docx]

Table S3 BLASTX results of genes involved in plant hormone signaling, flower development and cell division against TAIR.

| Gene name | Abbreviation | Contig | TAIR Blast Top Hit | |
| --- | --- | --- | --- | --- |
|  |  |  | Locus | Description |
| *Jatropha ABA DEFICIENT 1* | *JcABA1* | Contig3670 | AT5G67030 | Encodes a single copy zeaxanthin epoxidase gene that functions in first step of the biosynthesis of the abiotic stress hormone abscisic acid (ABA) |
| *Jatropha ABA DEFICIENT 2* | *JcABA2* | Contig23469 | AT1G52340 | Encodes a cytosolic short-chain dehydrogenase/reductase involved in the conversion of xanthoxin to ABA-aldehyde during ABA biosynthesis. |
| *Jatropha ABRUPTUS* | *JcABR* | Contig6499 | AT2G34650 | Encodes a protein serine/threonine kinase that may act as a positive regulator of cellular auxin efflux, as a binary switch for PIN polarity, and as a negative regulator of auxin signaling. |
| *Jatropha AGAMOUS* | *JcAG* | Contig13323 | AT4G18960 | Floral homeotic gene encoding a MADS domain transcription factor. |
| *Jatropha AGAMOUS-LIKE* | *JcAGL* | Contig13644 | AT3G58780 | Control dehiscence zone differentiation and promote the lignification of adjacent cells. |
| *Jatropha AINTEGUMENTA* | *JcANT* | Contig15354 | AT4G37750 | ANT is required for control of cell proliferation and encodes a putative transcriptional regulator similar to AP2. |
| *Jatropha APETALA 1* | *JcAP1* | Contig3340 | AT1G69120 | Floral homeotic gene encoding a MADS domain protein homologous to SRF transcription factors. |
| *Jatropha APETALA 3* | *JcAP3* | Contig17526 | AT3G54340 | Floral homeotic gene encoding a MADS domain protein homologous to SRF transcription factors. Specifies petal and stamen identities. |
| *Jatropha auxin response factor 1* | *JcARF1* | Contig5533 | AT1G59750 | Encodes a member of the auxin response factor family. ARFs bind to the *cis* element 5'-TGTCTC-3' ARFs mediate changes in gene expression in response to auxin. |
| *Jatropha auxin response factor 5* | *JcARF5* | Contig4371 | AT1G19850 | Encodes a transcription factor mediating embryo axis formation and vascular development. Similar toARF1 shown to bind to auxin responsive elements (AREs). |
| *Jatropha AUXIN-RESISTANT1* | *JcAUX1* | Contig3566 | AT2G38120 | Encodes an auxin influx transporter. AUX1 resides at the apical plasma membrane of protophloem cells and at highly dynamic subpopulations of Golgi apparatus. |
| *Jatropha BRI1-ASSOCIATED RECEPTOR KINASE 1* | *JcBAK1* | Contig5907 | AT4G33430 | Leu-rich receptor Serine/threonine protein kinase. Component of BR signaling that interacts with BRI1 in vitro and in vivo to form a heterodimer. |
| *Jatropha BRASSINOSTEROID*  *-INSENSITIVE 1* | *JcBRI1* | Contig672 | AT4G39400 | Encodes a plasma membrane localized leucine-rich repeat receptor kinase involved in brassinosteroid signal transduction. |
| *Jatropha BRASSINOSTEROID -SIGNALING KINASE 8* | *JcBSK8* | Contig10242 | AT5G41260 | Protein kinase protein with tetratricopeptide repeat domain; FUNCTIONS IN: binding, protein kinase activity, kinase activity, ATP binding. |
| *Jatropha BRASSINAZOLE RESISTANT 1* | *JcBZR1* | Contig3526 | AT1G75080 | Encodes a positive regulator of the brassinosteroid (BR) signaling pathway that mediates both downstream BR responses and negative feedback regulation of BR biosynthesis. |
| *Jatropha calcium-dependent*  *protein kinase 4* | *JcCDPK4* | Contig370 | AT4G09570 | Encodes a member of Calcium Dependent Protein Kinase (CDPK) gene family. Positive regulator of ABA signaling. |
| *Jatropha cytokinin oxidases/dehydrogenase 1* | *JcCKX1* | Contig11550 | AT2G41510 | Encodes a protein whose sequence is similar to cytokinin oxidase/dehydrogenase, which catalyzes the degradation of cytokinins. |
| *Jatropha cytokinin oxidases/dehydrogenase 4* | *JcCKX4* | Contig13020 | AT4G29740 | Encodes a protein whose sequence is similar to cytokinin oxidase/dehydrogenase, which catalyzes the degradation of cytokinins. |
| *Jatropha cytokinin oxidases/dehydrogenase 5* | *JcCKX5* | Contig1991 | AT1G75450 | Encodes a protein whose sequence is similar to cytokinin oxidase/dehydrogenase, which catalyzes the degradation of cytokinins. |
| *Jatropha CLAVATA1* | *JcCLV1* | Contig615 | AT5G65700 | Encodes a CLAVATA1-related receptor kinase-like protein required for both shoot and flower meristem function. |
| *Jatropha CORONATINE INSENSITIVE 1* | *JcCOI1* | Contig19285 | AT2G39940 | Encodes a protein containing Leu-rich repeats and a degenerate F-box motif. |
| *Jatropha CONSTANS-LIKE2* | *JcCOL2* | Contig2841 | AT3G02380 | Homologous to the flowering-time gene CONSTANS (CO) encoding zinc-finger proteins. |
| *Jatropha cyclin A3;2* | *JcCycA3;2* | Contig15009 | AT1G47210 | Cyclin-dependent protein kinase 3;2 (CycA3;2). |
| *Jatropha cyclin D3;1* | *JcCycD3;1* | Contig14149 | AT4G34160 | Encodes a cyclin D-type protein involved in the switch from cell proliferation to the final stages of differentiation. The gene is transcriptionally regulated by cytokinin and brassinosteroid. |
| *Jatropha cyclin D3;2* | *JcCycD3;2* | Contig2870 | AT5G67260 | Encode CycD3;2. Important for determining cell number in developing lateral organs. Mediating cytokinin effect in apical growth and development. |
| *Jatropha CYP89A5* | *JcCYP89A5* | Contig17235 | AT1G64950 | Cytochrome P450 superfamily protein. |
| *Jatropha EIN3-binding*  *F-box 1* | *JcEBF1* | Contig15749 | AT2G25490 | Encodes an F-box protein involved in the ubiquitin/proteasome-dependent proteolysis of EIN3. |
| *Jatropha ENHANCED*  *DISEASE SUSCEPTIBILITY 5* | *JcEDS5* | Contig19644 | AT4G39030 | Encodes an orphan multidrug and toxin extrusion transporter. Essential component of salicylic acid-dependent signaling for disease resistance. |
| *Jatropha ethylene-insensitive*  *protein 2* | *JcEIN2* | Contig17766 | AT5G03280 | Involved in ethylene signal transduction. Acts downstream of CTR1. Positively regulates ORE1 and negatively regulates mir164A, B, C to regulate leaf senescence. |
| *Jatropha ethylene*  *insensitive protein 3* | *JcEIN3* | Contig16976 | AT3G20770 | Encodes EIN3, a nuclear transcription factor that initiates downstream transcriptional cascades for ethylene responses. |
| *Jatropha ethylene-responsive transcription factor 1* | *JcERF1* | Contig3773 | AT4G17500 | Encodes a member of the ethylene response factor subfamily B-3 of ERF/AP2 transcription factor family (ATERF-1). |
| *Jatropha ethylene receptor 1* | *JcETR1* | Contig959 | AT1G66340 | Similar to prokaryote sensory transduction proteins. Contains a histidine kinase and a response regulator domain. |
| *Jatropha GA20 oxidase* | *JcGA20ox* | Contig22330 | AT5G51810 | Encodes gibberellin 20-oxidase. Involved in gibberellin biosynthesis. |
| *Jatropha GA*  *INSENSITIVE DWARF1* | *JcGID1* | Contig19801 | AT5G27320 | Encodes a gibberellin (GA) receptor ortholog of the rice GA receptor gene (OsGID1). Interacts with DELLA proteins in vivo in the presence of GA4. |
| *Jatropha GIGANTEA* | *JcGI* | Contig5489 | AT1G22770 | GI promotes flowering under long days in a circadian clock-controlled flowering pathway. |
| *Jatropha Histone H4* | *JcHistone H4* | Contig17440 | AT1G07660 | Histone superfamily protein. |
| *Jatropha histidine kinase 2* | *JcHK2* | Contig18628 | AT2G01830 | Histidine kinase: cytokinin-binding receptor that transduces cytokinin signals across the plasma membrane. |
| *Jatropha histidine kinase 3* | *JcHK3* | Contig5427 | AT1G27320 | Histidine kinase, a cytokinin receptor that controls cytokinin-mediated leaf longevity through a specific phosphorylation of the response regulator, ARR2. |
| *Jatropha histidine kinase 4* | *JcHK4* | Contig9387 | AT2G01830 | Histidine kinase: cytokinin-binding receptor that transduces cytokinin signals across the plasma membrane. |
| *Jatropha histidine*  *phosphotransfer protein 1* | *JcHP1* | Contig1888 | AT3G21510 | Encodes AHP1, function as redundant positive regulators of cytokinin signaling. |
| *Jatropha histidine*  *phosphotransfer protein 5* | *JcHP5* | Contig3168 | AT1G03430 | Encodes AHP5. |
| *Jatropha INDOLEACETIC ACID-INDUCED PROTEIN 14* | *JcIAA14* | Contig159 | AT4G14550 | A member of the Aux/IAA protein family. |
| *Jatropha isopentenyl*  *transferase 19* | *JcIPT9* | Contig7878 | AT5G20040 | Encodes tRNA isopentenyltransferase. |
| *Jatropha jasmonate*  *ZIM-domain (JAZ) protein 1* | *JcJAZ1* | Contig7545 | AT1G19180 | A nuclear-localized protein involved in jasmonate signaling. JAZ transcript levels rise in response to a jasmonate stimulus. |
| *Jatropha JASMONIC ACID CARBOXYL*  *METHYLTRANSFERASE* | *JcJMT* | Contig4495 | AT1G19640 | Encodes an enzyme that catalyzes the formation of methyljasmonate from jasmonic acid. Its expression is induced in response to wounding or methyljasmonate treatment. |
| *Jatropha KNOTTED-LIKE HOMEOBOX* | *JcKNOX* | Contig13732 | AT4G08150 | A member of class I knotted1-like homeobox gene family (together with KNAT2). Similar to the knotted1 (kn1) homeobox gene of maize. |
| *Jatropha LEAFY* | *JcLFY* | Contig21406 | AT5G61850 | Encodes transcriptional regulator that promotes the transition to flowering. Involved in floral meristem development. |
| *Jatropha LEUNIG* | *JcLUG* | Contig22412 | AT4G32551 | Regulates floral organ identity, gynoecium and ovule development. Negatively regulates AG. |
| *Jatropha LONELY GUY 3* | *JcLOG3* | Contig16485 | AT2G37210 | Encodes a protein of unknown function. It has been crystallized and shown to be structurally almost identical to the protein encoded by At5g11950. |
| *Jatropha LONELY GUY 7* | *JcLOG7* | Contig19304 | AT5G06300 | Putative lysine decarboxylase family protein. |
| *Jatropha LONELY GUY 8* | *JcLOG8* | Contig10713 | AT5G11950 | It has been crystallized and shown to be structurally almost identical to the protein encoded by At2G37210. |
| *Jatropha LONELY GUY 9* | *JcLOG9* | Contig1943 | AT1G50575 | Putative lysine decarboxylase family protein. |
| *Jatropha mitogen-activated protein kinase 6* | *JcMPK6* | Contig8387 | AT2G43790 | Encodes a MAP kinase induced by pathogens, ethylene biosynthesis, oxidative stress and osmotic stress. Also involved in ovule development. |
| *Jatropha PATHOGENESIS -RELATED GENES 4* | *JcNPR4* | Contig9705 | AT4G19660 | Encodes NPR4, ankyrin repeat BTB/POZ domain-containing protein. |
| *Jatropha OXOPHYTODIENOATE -REDUCTASE 3* | *JcOPR3* | Contig7789 | AT2G06050 | Encodes a 12-oxophytodienoate reductase that is required for jasmonate biosynthesis. |
| *Jatropha PHOSPHOLIPASED ALPHA 2* | *JcPLD2* | Contig2488 | AT1G52570 | Member of C2-PLD subfamily. |
| *Jatropha protein phosphatase 2C* | *JcPP2C* | Contig10109 | AT2G30020 | Encodes AP2C1. Belongs to the clade B of the PP2C-superfamily. Acts as a MAPK phosphatase that negatively regulates MPK4 and MPK6. |
| *Jatropha PYR1-LIKE 1* | *JcPYL1* | Contig804 | AT5G46790 | PYR/PYL/RCAR family proteins function as abscisic acid sensors. Mediate ABA-dependent regulation of protein phosphatase 2Cs ABI1 and ABI2. |
| *Jatropha PYR1-LIKE 4* | *JcPYL4* | Contig6470 | AT2G38310 | Abscisic acid sensors. |
| *Jatropha PYR1-LIKE 8* | *JcPYL8* | Contig19419 | AT5G53160 | Abscisic acid sensors. |
| *Jatropha REPRESSOR OF ga1-1* | *JcRGA1* | Contig6692 | AT2G01570 | Putative transcriptional regulator repressing the gibberellin response and integration of phytohormone signaling. |
| *Jatropha RGA LIKE 2* | *JcRGL2* | Contig1527 | AT3G03450 | Encodes a DELLA protein, a member of the GRAS superfamily of putative transcription factors. |
| *Jatropha RECEPTOR-LIKE PROTEIN KINASE 2* | *JcRPK2* | Contig2457 | AT3G02130 | Encodes a receptor-like kinase RPK2 (also known as TOADSTOOL 2/TOAD2). Functions as a regulator of meristem maintenance. |
| *Jatropha type-B*  *response regulator 2* | *JcRRB2* | Contig19198 | AT4G16110 | Type B response regulator. |
| *Jatropha type-A*  *response regulator 3* | *JcRRA3* | Contig16708 | AT1G59940 | Type A response regulator. |
| *Jatropha type-A*  *response regulator 5* | *JcRRA5* | Contig12959 | AT3G48100 | Type A response regulator. |
| *Jatropha type-A*  *response regulator 9* | *JcRRA9* | Contig12946 | AT3G57040 | Type A response regulator. |
| *Jatropha type-A*  *response regulator 17* | *JcRRA17* | Contig22202 | AT3G56380 | Type A response regulator. |
| *Jatropha type-B*  *response regulator 18* | *JcRRB18* | Contig18864 | AT5G58080 | Type B response regulator. |
| *Jatropha small*  *auxin up RNA* | *JcSAUR* | Contig9451 | AT4G38840 | SAUR-like auxin-responsive protein family. |
| *Jatropha SEPALLATA 1* | *JcSEP1* | Contig11701 | AT5G15800 | Encodes a MADS box transcription factor involved flower and ovule development. |
| *Jatropha SEPALLATA 2* | *JcSEP2* | Contig3088 | AT5G15800 | Encodes a MADS box transcription factor involved flower and ovule development. |
| *Jatropha SEPALLATA 3* | *JcSEP3* | Contig3464 | AT1G24260 | Encodes a MADS box transcription factor. |
| *Jatropha SEUSS* | *JcSEU* | Contig7056 | AT1G43850 | Encodes a transcriptional co-regulator of AG that functions with LEU to repress AG in the outer floral whorls. |
| *Jatropha SHOOT MERISTEMLESS* | *JcSMT* | Contig8878 | AT1G62360 | Class I knotted-like homeodomain protein that is required for shoot apical meristem (SAM) formation during embryogenesis and for SAM function throughout the lifetime of the plant. |
| *Jatropha SUPPRESSOR OF OVEREXPRESSION OF CO1* | *JcSOC1* | Contig12937 | AT2G45660 | Controls flowering and is required for CO to promote flowering. It acts downstream of FT. |
| *Jatropha SPINDLY* | *JcSPY* | Contig22126 | AT3G11540 | Encodes an N-acetyl glucosamine transferase that may glycosylate other molecules involved in GA signaling. |
| *Jatropha TRANSPORT INHIBITOR RESPONSE1* | *JcTIR1* | Contig20392 | AT3G62980 | Encodes an auxin receptor that mediates auxin-regulated transcription. |
| *Jatropha TSO1* | *JcTSO1* | Contig17984 | AT3G22780 | Putative DNA binding protein (TSO1). |
