# Supplementary material for: Transcriptome of the inflorescence meristems of the biofuel plant Jatropha curcas treated with cytokinin
Source: BMC Genomics. 2014 Nov 17;15(1):974. doi: 10.1186/1471-2164-15-974 (PMC4246439; doi:10.1186/1471-2164-15-974)
Supplement: Supplementary file 6 — Additional file 6: Table S5: Primer sequences and PCR amplicon lengths of the selected genes. (DOCX 18 KB) [file 12864_2014_6670_MOESM6_ESM.docx]

Table S5 Primer sequences and PCR amplicon lengths of the selected genes.

| Gene name | Forward | Reverse | Product size (bp) |
| --- | --- | --- | --- |
| JcBZR1 | TGCCAGTCCAACATCCTCTTCATT | GGGAAGAAAGCGGTGGAGTTACAG | 161 |
| JcCOL2 | GACAGGGAAGCAAGGGTCCTAAGAT | CCTTCCTTGACGCATACCTGATTGT | 85 |
| JcCycA3;2 | GAGGGATTCTGGTGGATTGGTTG | AGGGCTGATTTCTTCGTACTTTGAT | 185 |
| JcCycD3;1 | GCTTGTCTTGTTGAAAATAAGGGCT | TTGCTCGTTTAGATGCTGTGGAAT | 132 |
| JcCycD3;2 | GTTTGTAGTGAAATTCGTGGTCAGA | TAGCCTCTTTACGAGCAACCATT | 187 |
| JcCYP89A5 | TCGCCCACCAAACTCTAATCCAA | GGTGGAGGATTTCGGAAGTGAGG | 165 |
| JcEIN3 | GCAGTTGGAGGAGTGCCACAGGAT | GTTACGGATGGACAATGAACGCAGG | 175 |
| JcGI | TGGGTAAGCGTATTGCCGGGTTC | GCGGTAGGTTGAGTTTCGGGTGT | 199 |
| JcHK2 | TAGCGAGAAACTGGGATTTAGCA | TTCGTGATTGGCTTATTAGTGGG | 144 |
| JcHK3 | AACAGAATTAGCCAGAAGGAACAGT | CGAACAAGAAGTATGGGATAGGGA | 112 |
| JcHK4 | ATTCTCATTCACTGCTGCTTTCGG | AGCCTCTTCAAATGGTATCTGGTTA | 161 |
| JcHP1 | CCAAAATCCCATTCTACTTGCATCA | TGCGATGAGAAGAATACTGAAGGGT | 169 |
| JcHP5 | TAGTTCCAGCATGGGTGCAGCGA | TCTGTTGCTCCAGCCGGAATAAGG | 167 |
| JcIAA14 | CGTTAGCGAGCGAGATTCACCAGG | ACGCTTGCCGGTATCTGACACCAC | 91 |
| JcJAZ1 | GTGCTACCGTTTCCACTTCCCTCC | GAGCTAGGTCACCGGAGAAGTCGA | 136 |
| JcLFY | TCGTTTAGCCATTTGGTATGTTCC | CAAGCCATTCATCCTAACCTACAAC | 181 |
| JcRRA17 | TCCTAAGCCTAAATACTCCAATGC | GGATAAGATATTGGGAGACTATGGAG | 163 |
| JcRRA3 | CAGTTGCTGCTCATTTCATTCGTC | ATCTCCTTCGCCGTCGCCTTTA | 104 |
| JcRRA5 | AAGTTACTGCTGTTGAGAGCGGGA | TCTGAAAGCTGATGATTCCTTGATC | 176 |
| JcRRA9 | GTTGCGGTTGTTTCTGCTGTTGTG | AAGATGTTTAGAGGAAGGAGCAGAGG | 185 |
| JcRRB18 | TCTGGTTCAATGTACCCACAACGC | GGTGGGATGAACATAGCCAGGATTA | 105 |
| JcRRB2 | GCTACAGGTCAAATACCAGCACAA | CTGTTGCCCATCGCCAAATCTCA | 157 |
| JcTSO1 | GGGCAATACAACTAAGATACAAGGG | CATCAGCAATGACAGAATCCTCC | 208 |
